# Methionine Metabolism Dictates PCSK9 Expression and Antitumor Potency of PD‐1 Blockade in MSS Colorectal Cancer

**DOI:** 10.1002/advs.202501623

**Published:** 2025-03-24

**Authors:** Qi‐Long Wang, Zijie Chen, Xiaofei Lu, Huizhen Lin, Huolun Feng, Nuozhou Weng, Liwen Chen, Mengnan Liu, Li Long, Lingjun Huang, Yongmei Deng, Kehong Zheng, Xiaojun Zheng, Yong Li, Ting Cai, Jiabin Zheng, Wei Yang

**Affiliations:** ^1^ Medical Research Institute Guangdong Provincial People's Hospital (Guangdong Academy of Medical Sciences) Southern Medical University Guangzhou 510080 China; ^2^ Guangdong Provincial Key Laboratory of Molecular Oncologic Pathology Department of Pathology School of Basic Medical Sciences Southern Medical University Guangzhou 510515 China; ^3^ Department of Gastrointestinal Surgery Department of General Surgery Guangdong Provincial People's Hospital (Guangdong Academy of Medical Sciences) Southern Medical University Guangzhou 510080 China; ^4^ Department of General Surgery Zhujiang Hospital Southern Medical University Guangzhou 510280 China

**Keywords:** immunotherapy resistance, methionine metabolism, MSS colorectal cancer, PCSK9

## Abstract

Nutrient metabolisms are vitally interrelated to cancer progression and immunotherapy. However, the mechanisms by which nutrient metabolisms interact to remodel immune surveillance within the tumor microenvironment remain largely unexplored. Here it is demonstrated that methionine restriction inhibits the expression of proprotein convertase subtilisin/kexin type 9 (PCSK9), a key regulator of cholesterol homeostasis and a potential target for cancer immunotherapy, in colorectal cancer (CRC) but not in the liver. Mechanistically, methionine is catabolized to S‐adenosylmethionine (SAM), promoting mRNA transcription of PCSK9 through increased DNA methyltransferase 1 (DNMT1)‐mediated DNA methylation and suppression of sirtuin 6 (SIRT6) expression. Furthermore, both PCSK9 inhibition and dietary methionine restriction (DMR) potentiate PD‐1 blockade therapy and foster the infiltration of CD8^+^ T cells in Colon 26 tumor‐bearing mice—a proficient mismatch repair (pMMR)/microsatellite stable (MSS) CRC model that exhibits limited response to anti‐PD‐1 therapy. Moreover, combining 5‐fluorouracil (5‐FU) chemotherapy with PCSK9 inhibition and PD‐1 blockade further augments therapeutic efficacy for MSS CRC. The findings establish a mechanistic link between amino acid metabolism and cholesterol metabolism within the tumor microenvironment where tumor cells sense methionine to regulate PCSK9 expression, highlighting promising combination therapeutic strategies that may greatly benefit MSS CRC patients.

## Graphical abstract

This study establishes a mechanistic link between amino acid metabolism and cholesterol metabolism within the tumor microenvironment. Mechanistically, methionine is catabolized to S‐adenosyl‐methionine (SAM) to promote the transcription of PCSK9, a key orchestrator of cholesterol metabolism, via DNMT1‐mediated DNA methylation and suppressing SIRT6 expression, leading to the ectopic elevation of PCSK9 in tumor and immunotherapy resistance.

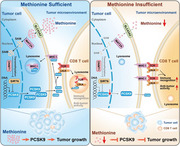

## 1 Introduction

Nutrients and their derivatives within the tumor microenvironment are inextricably linked to tumor progression and immune evasion.^[^ ^1^, ^2^ ^]^ Tumor cells competitively seize the amino acids in the microenvironment to fulfill their voracious proliferation, generating an undernourished microenvironment marked by a deficiency in arginine, asparagine, glutamine, methionine, and tryptophan, which restrains the survival, differentiation, and function of immune cells.^[^ ^3^, ^4^, ^5^, ^6^, ^7^, ^8^, ^9^ ^]^ The immunosuppressive microenvironment constituted by tumor‐associated amino acid derivatives, such as kynurenine and spermidine, also gives rise to immune cell dysfunction and facilitates tumor immune evasion.^[^ ^10^, ^11^ ^]^ Furthermore, tumor cells and tumor‐associated macrophages consume most of the environmental cholesterol, an essential component of cellular membranes, resulting in cholesterol deficiency in tumor‐infiltrating CD8^+^ T cells, and subsequently exhaustion and dysfunction.^[^ ^12^, ^13^ ^]^ Hence, targeting amino acid metabolism and cholesterol metabolism in the tumor microenvironment for cancer therapy have been of increasing attention.^[^ ^12^, ^14^, ^15^, ^16^ ^]^ Nevertheless, how tumor cells coordinate these nutrient metabolisms to reshape the immune surveillance in the tumor microenvironment remains largely unknown.

Proprotein convertase subtilisin/kexin type 9 (PCSK9), a key orchestrator of cholesterol homeostasis by reducing low‐density lipoprotein receptor (LDLR) recycling to cell membrane of hepatocytes,^[^ ^17^, ^18^, ^19^ ^]^ is aberrantly elevated in tumor cells and involved in tumor progression.^[^ ^20^, ^21^, ^22^, ^23^, ^24^ ^]^ Specifically, PCSK9 promotes tumor cell proliferation and inhibits apoptosis in neuroglioma, lung adenocarcinoma, melanoma, hepatocellular carcinoma, and colorectal cancer.^[^ ^22^, ^23^, ^24^, ^25^ ^]^ Moreover, PCSK9 suppresses immune recognition of tumor cells by directly binding to major histocompatibility complex type I (MHC I) for lysosome localization and degradation,^[^ ^20^ ^]^ and impairs anti‐tumor immunity by preventing LDLR‐mediated T cell receptor (TCR) recycling and signaling of CD8^+^ T cell,^[^ ^21^ ^]^ thus achieving immune evasion. Owing to the multifaceted role of PCSK9 in tumor microenvironment, it is emerging as a crucial immunometabolism checkpoint and a promising target for cancer treatment.^[^ ^26^, ^27^ ^]^ Therefore, significant attention should be devoted to elucidating how microenvironment metabolism contributes to tumor cell PCSK9 expression.

In this study, a specific regulatory mechanism of PCSK9 expression in colorectal cancer (CRC) tumor cells was discovered, wherein methionine catabolism promotes mRNA transcription of PCSK9 by facilitating DNA methylation mediated by DNA methyltransferase 1 (DNMT1) and suppressing sirtuin 6 (SIRT6) expression. Both dietary methionine restriction (DMR) and PCSK9 inhibition sensitized microsatellite stable (MSS) CRC to PD‐1 blockade. Furthermore, the combination of PCSK9 inhibition and PD‐1 blockade along with 5‐fluorouracil (5‐FU) chemotherapy suppressed tumor growth in MSS CRC.

## 2 Results

### 2.1 Methionine Deprivation Inhibits Colorectal Cancer (CRC) Proprotein Convertase Subtilisin/Kexin Type 9 (PCSK9) Expression

**Figure 1:**
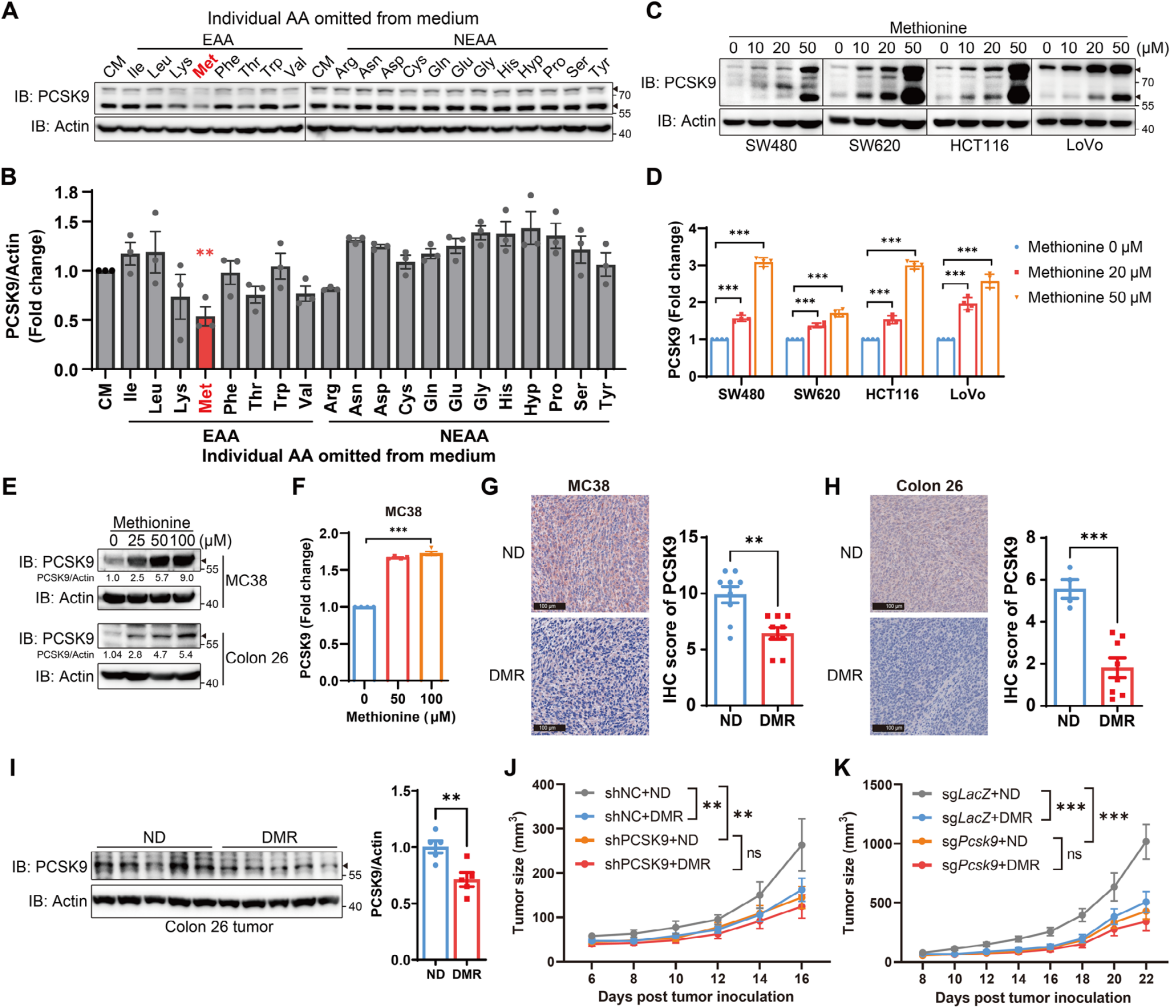
Methionine deprivation inhibits PCSK9 expression in vivo. A) Immunoblotting analysis of PCSK9 in SW480 cells cultured with RPMI 1640 medium without individual amino acids for 6h. B) Densitometric quantification of the ratio of PCSK9 to β‐actin (*n* = 3). The ratio in complete medium group was set as 1. C–F) The expression and secretion of PCSK9 in both human and mouse CRC cells with methionine deprivation for 6 h and then methionine supplementation for 24h. Immunoblotting analysis of PCSK9 in human CRC cell lines (SW480, SW620, HCT116, and LoVo) C) and mouse CRC cell lines (MC38 and Colon 26) E). The densitometric quantification of the ratio of PCSK9 to β‐actin is calculated E). ELISA analysis of PCSK9 in culture supernatant of human CRC cell lines (SW480, SW620, HCT116, and LoVo) D) and mouse CRC cell lines (MC38 and Colon 26) F). G,H) Immunohistochemistry analysis of PCSK9 in tumors from MC38‐ (G, *n* = 9) and Colon 26‐ (H, *n* = 4 (ND), *n* = 8 (DMR)) bearing mice fed with normal diet and methionine restriction diet (left). The abundance of PCSK9 was assessed (right). I) Immunoblotting analysis of PCSK9 in tumors from Colon 26‐bearing mice fed with normal diet and methionine restriction diet. Densitometric quantification of the ratio of PCSK9 to β‐actin is calculated. J,K) Tumor volume of PCSK9‐KD (J, *n* = 10) or PCSK9‐KO (K, *n* = 9) MC38‐bearing mice fed with normal diet and methionine restriction diet. EAA, essential amino acid; NEEA, non‐essential amino acid; CM, complete medium; ND, normal diet; DMR, dietary methionine restriction. Data were analyzed by unpaired two‐tailed Student's *t*‐test B, D, F, G, and H) or two‐way ANOVA J and K). Error bars denote for the s.e.m. ns, not significant; **p < 0.01, ***p < 0.001.

Amino acid metabolism plays a crucial role in tumor progression and the response to therapy.^[^ ^14^, ^15^, ^28^ ^]^ To determine whether abnormal amino acid metabolism is related to alterations of PCSK9 expression in tumor cells, we cultured primary tumor‐derived and metastasis‐derived human CRC cells (SW480 and SW620 cells) in a medium devoid of individual amino acids. The results showed that deprivation of methionine resulted in the most pronounced downregulation of PCSK9 (**Figure** **1**A,B; Figure S1A, Supporting Information). Furthermore, supplementation of methionine also increased the expression and secretion of PCSK9 in both human and mouse CRC cells (Figure 1C–F). To further establish whether PCSK9 expression is regulated by methionine in vivo, we examined PCSK9 levels in tumors from syngeneic mouse hosts inoculated with mouse CRC cell lines (MC38 and Colon 26) and fed with a methionine restriction diet. Consistent with the previously reported results, tumor growth was significantly alleviated by DMR (Figure S1B–E, Supporting Information).^[^ ^29^, ^30^, ^31^ ^]^ Moreover, dietary methionine restriction (DMR) significantly reduced the expression of PCSK9 in CRC tumors (Figure 1G–I; Figure S1F, Supporting Information).

Nevertheless, the deprivation of lysine or threonine also resulted in a slight reduction in PCSK9 expression (Figure 1A,B234; Figure S1A, Supporting Information). Therefore, we examined the effect of lysine and threonine supplementation on PCSK9 expression. Indeed, the addition of these amino acids also led to a significant upregulation of PCSK9 expression (Figure S1G, Supporting Information), similar to that observed with methionine supplementation. Together these findings indicate an inextricable relationship between amino acid metabolism and cholesterol metabolism.

Given that PCSK9 is mainly expressed in the liver and secreted into the blood, we also detected the PCSK9 levels in the liver and blood from a CRC mouse model fed with a methionine restriction diet. Interestingly, the PCSK9 levels in liver and blood did not decline upon DMR (Figure S1H,I, Supporting Information). Furthermore, methionine supplementation did not modify the expression of PCSK9 in liver cells (Figure S1J, Supporting Information). Indeed, the expression of sterol regulatory element binding transcription factor 2 (SREBP2) and hepatocyte nuclear factor 1 alpha (HNF1α), two of the most important regulators for PCSK9 transcription,^[^ ^32^, ^33^ ^]^ also remain unaltered in liver cells upon methionine supplementary (Figure S1K,L, Supporting Information). Moreover, knockout of SREBP2 or HNF1α in colorectal cancer cells could not inhibit the expression of PCSK9 induced by methionine (Figure S1M–P, Supporting Information). Therefore, these results indicated that methionine specifically regulates the expression of PCSK9 in CRC tumors through a mechanism different from that in the liver.

Furthermore, to assess the role of PCSK9 on DMR‐mediated tumor growth inhibition, we knocked down/out the PCSK9 in MC38 cell lines. Consistent with previous studies, knockdown/knockout of PCSK9 attenuated tumor growth.^[^ ^20^, ^21^ ^]^ However, DMR did not further alleviate tumor progression in PCSK9‐KD/KO MC38 inoculated mice (Figure 1J,K).

Taken together, these results demonstrated that tumor PCSK9 expression is modulated by methionine alterations both in vitro and in vivo, and participates in DMR‐regulated CRC tumor progression.

### 2.2 Methionine Promotes PCSK9 mRNA Transcription

**Figure 2:**
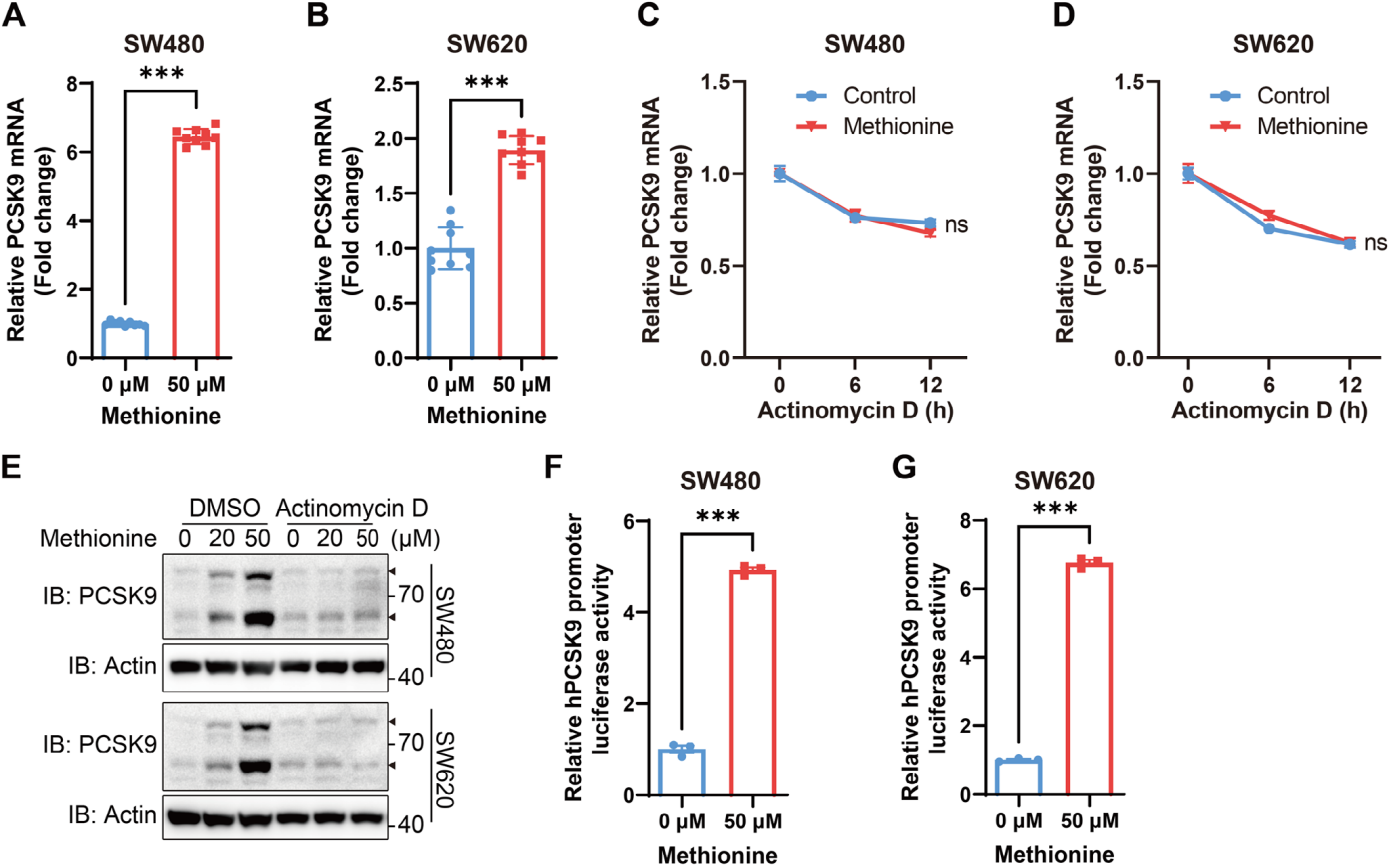
Methionine promotes PCSK9 mRNA transcription. A,B) Relative mRNA level of PCSK9 in SW480 (A, *n* = 9) and SW620 (B, *n* = 9) cells with methionine deprivation for 6 h and then methionine supplementation (0, 50 µm) for 24h. C,D) Relative mRNA level of PCSK9 in SW480 (C, *n* = 9) and SW620 (D, *n* = 9) cells with methionine deprivation for 6 h and then methionine supplementation (0, 50 µm) together with actinomycin D (10 µg mL^−1^) treatment for the indicated time were analyzed. The mRNA stability of PCSK9 was assessed. E) Immunoblotting analysis of PCSK9 in SW480 (upper panel) and SW620 (lower panel) cells with methionine deprivation for 6 h and then methionine supplementation (0, 50 µm) together with actinomycin D (10 µg mL^−1^) treatment for 24h. F,G) Luciferase reporter assays of hPCSK9 promoter activity in SW480 (F, *n* = 3) and SW620 (G, *n* = 3) cells transfected with hPCSK9 promoter luciferase reporter plasmid together with Renilla luciferase plasmid, and then cultured with methionine deprivation for 6 h and supplementation (0, 50 µm) for 24h. The relative hPCSK9 promoter luciferase activity for methionine starvation group was set as 1. Data were analyzed by unpaired two‐tailed Student's *t*‐test A, B, F, and G) or two‐way ANOVA C and D). Error bars denote for the s.e.m. ns, not significant; ***p < 0.001.

Protein expression levels are primarily regulated through mRNA transcription and stability, translation, and protein stability.^[^ ^34^ ^]^ To investigate how methionine deprivation regulates PCSK9, we evaluated the protein stability and mRNA transcription of PCSK9 in CRC cells. Methionine deprivation had no impact on PCSK9 protein stability (Figure S2A,B, Supporting Information). However, the level of PCSK9 mRNA increased when methionine was supplemented (**Figure** **2**A,B; Figure S2C,D, Supporting Information). Furthermore, methionine supplementation increased the mRNA transcription of PCSK9 in CRC tumor cell line but not in liver cell line (Figure S2E,F, Supporting Information). Nevertheless, the mRNA stability of PCSK9 remained unchanged upon methionine deprivation (Figure 2C,D). Together, these results implied a possibility that methionine regulates PCSK9 mRNA transcription. To test this hypothesis, we detected PCSK9 expression while treated with actinomycin D, a transcription inhibitor. The result showed that blocking mRNA transcription prevented methionine‐induced upregulation of PCSK9 (Figure 2E). Moreover, methionine supplementation upregulated the promoter activity of *PCSK9* (Figure 2F,G). Thus, these results provided evidence that methionine promotes the mRNA transcription of PCSK9.

### 2.3 Methionine is Catabolized to S‐adenosylmethionine (SAM) to Promote PCSK9 Expression

**Figure 3:**
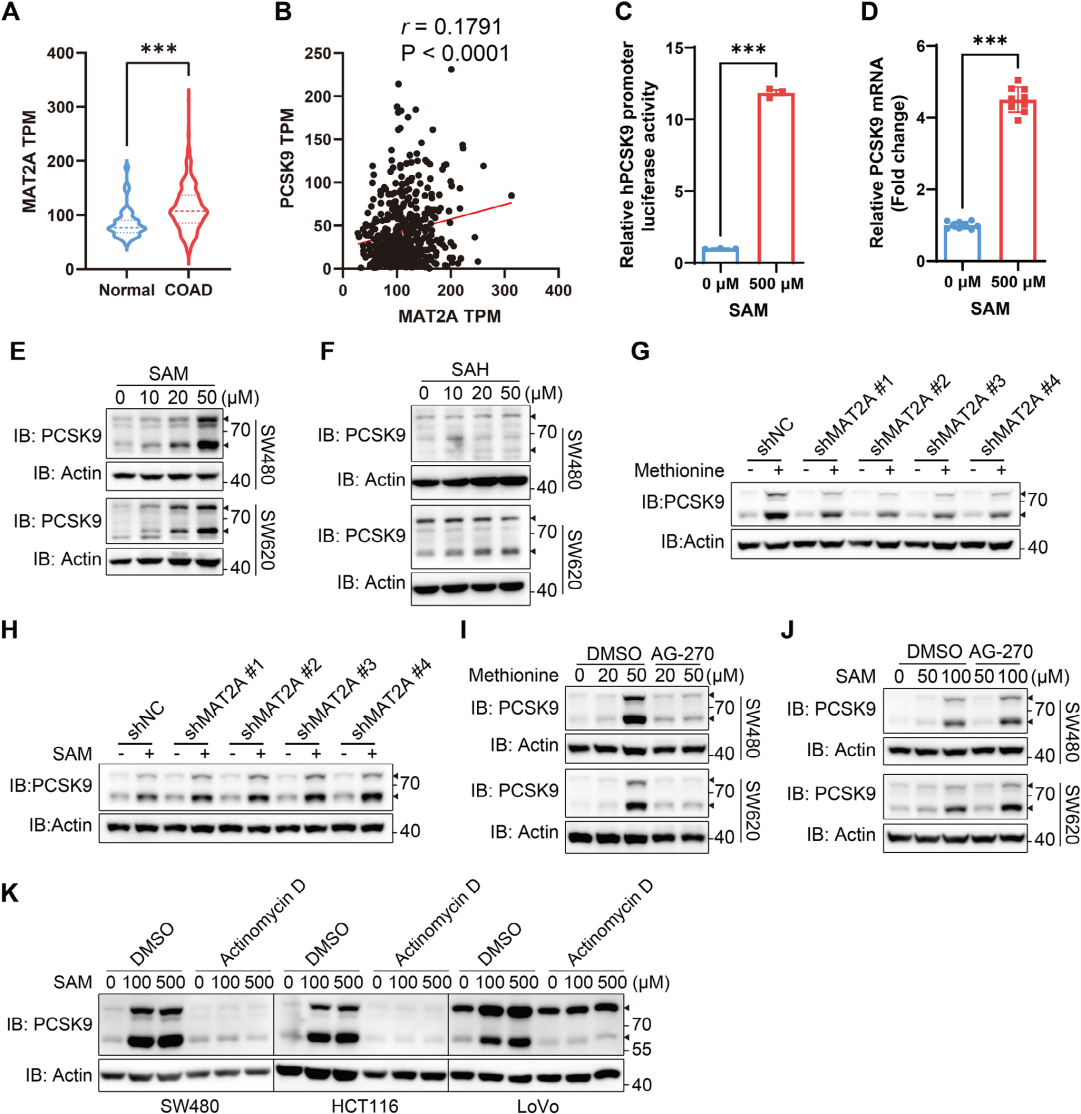
Methionine is catabolized to SAM to promote PCSK9 expression. A) The mRNA level of MAT2A in tumor tissues (*n* = 476) and normal tissues (*n* = 41) of Colon Adenocarcinoma from TCGA database. B) Correlation between MAT2A and PCSK9 mRNA levels in tumor tissues (*n* = 476) of Colon Adenocarcinoma from the TCGA database was calculated using linear regression. C) Luciferase reporter assays of hPCSK9 promoter activity in SW480 cells transfected with hPCSK9 promoter luciferase reporter plasmid together with Renilla luciferase plasmid, and then cultured with methionine deprivation for 6 h and SAM addition (0, 500 µm) for 24 h (*n* = 3). The relative hPCSK9 promoter luciferase activity for methionine starvation group was set as 1. D) Relative mRNA level of PCSK9 in SW480 cells with methionine deprivation for 6 h and then SAM addition (0, 500 µm) for 24 h (*n* = 9). E,F) Immunoblotting analysis of PCSK9 in SW480 (upper panel) and SW620 (lower panel) cells with methionine deprivation for 6 h and then SAM E) or SAH F) supplementation for 24h. G,H) Immunoblotting analysis of PCSK9 in SW480 cells transfected shMAT2A plasmids and treated with methionine G) or SAM H) for 24h. I,J) Immunoblotting analysis of PCSK9 in SW480 (upper panel) and SW620 (lower panel) cells with methionine deprivation for 6 h, and then SAM I) or SAH J) supplementation together with AG‐270 (1 µm) treatment for 24h. K) Immunoblotting analysis of PCSK9 in SW480, HCT116, and LoVo cells with methionine deprivation for 6 h and then SAM addition (0, 100, 500 µm) together with actinomycin D (10 µg mL^−1^) treatment for 24h. Data were analyzed by unpaired two‐tailed Student's *t*‐test (A, C, and D) or Pearson *r* (B). Error bars denote for the s.e.m. ***p < 0.001.

Methionine undergoes catabolism through a series of metabolic reactions.^[^ ^35^ ^]^ Once transported into cells, methionine is converted to the universal methyl donor S‐adenosylmethionine (SAM) under the catalysis of methionine adenosine transferases (MATs), and then to S‐adenosyl‐homocysteine (SAH), which is further metabolized to homocysteine by S‐adenosyl‐homocysteine hydrolase (AHCY) and ultimately to cysteine.^[^ ^14^, ^35^ ^]^ Indeed, the entry of methionine into cell is central to its regulatory effect on PCSK9 (Figure S3A,B, Supporting Information). Besides, using the RNA‐seq data of colon adenocarcinoma (COAD) cohort from TCGA database, we found that the expression of MAT2A, a rate‐limiting enzyme that primarily catalyzes the synthesis of SAM,^[^ ^36^ ^]^ was significantly higher in COAD tumor tissues than in adjacent normal tissues, and it was positively correlated with the expression of PCSK9 in tumor tissues (**Figure** **3**A,B). Although the expression of AHCY was also higher in tumor tissues than in adjacent normal tissues, it was not correlated with PCSK9 expression (Figure S3C,D, Supporting Information). Moreover, similar to methionine, SAM addition enhanced the promoter activity of *PCSK9* and mRNA transcription of PCSK9 (Figure 3C,D). Supplementation with SAM, but not SAH in the methionine‐depleted medium, increased PCSK9 expression (Figure 3E,F; Figure S3E,F, Supporting Information), indicating that conversion of methionine to SAM may be critical for PCSK9 expression. Furthermore, knocking down MAT2A or inhibiting MAT2A with AG‐270^[^ ^37^ ^]^ impeded the upregulation of PCSK9 induced by methionine, but not that induced by SAM (Figure 3G–J). Additionally, blocking mRNA transcription with actinomycin D also prevented SAM‐induced upregulation of PCSK9 (Figure 3K). Taken together, these observations suggested that methionine is catabolized to SAM to promote mRNA transcription of PCSK9.

### 2.4 DNA Methyltransferase 1 (DNMT1)‐Mediated Methylation Promotes mRNA Transcription of PCSK9

**Figure 4:**
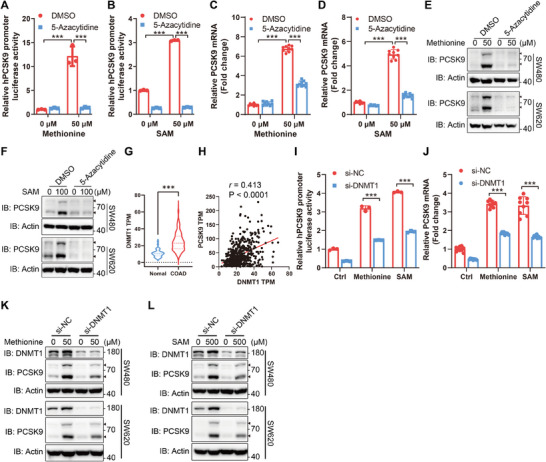
DNMT1‐mediated methylation promotes mRNA transcription of PCSK9. A,B) Luciferase reporter assays of hPCSK9 promoter activity in SW480 cells transfected with hPCSK9 promoter luciferase reporter plasmid together with Renilla luciferase plasmid, and then cultured with methionine deprivation for 6 h followed by methionine (A, *n* = 3) or SAM (B, *n* = 3) supplementation and 5‐azacytidine (5 µm) treatment for 24h. The relative hPCSK9 promoter luciferase activity for methionine starvation group was set as 1. C,D) Relative mRNA level of PCSK9 in SW480 cells with methionine deprivation for 6 h and then methionine (C, *n* = 9) or SAM (D, *n* = 6 or 9) supplementation together with 5‐azacytidine (5 µm) treatment for 24h. E,F) Immunoblotting analysis of PCSK9 in SW480 (upper panel) and SW620 (lower panel) cells with methionine deprivation for 6 h, and then methionine E) or SAM F) supplementation together with 5‐azacytidine (5 µm) treatment for 24h. G) The mRNA level of DNMT1 in tumor tissues (*n* = 476) and normal tissues (*n* = 41) of Colon Adenocarcinoma from TCGA database. H) Correlation between DNMT1 and PCSK9 mRNA levels in tumor tissues (*n* = 476) of Colon Adenocarcinoma from the TCGA database was calculated using linear regression. I) Luciferase reporter assays of hPCSK9 promoter activity in SW480 cells transfected with si‐DNMT1 (si‐NC served as a negative control) together with hPCSK9 promoter luciferase reporter plasmid and Renilla luciferase plasmid, and then cultured with methionine deprivation for 6 h followed by methionine or SAM supplementation for 24 h (*n* = 3). The relative hPCSK9 promoter luciferase activity for negative control was set as 1. J) Relative mRNA level of PCSK9 in SW480 cells transfected with si‐DNMT1 (si‐NC served as a negative control), and then cultured with methionine deprivation for 6 h and then methionine or SAM supplementation for 24 h (*n* = 9). K,L) Immunoblotting analysis of PCSK9 in SW480 (upper panel) and SW620 (lower panel) cells transfected with si‐DNMT1 (si‐NC served as a negative control), and then cultured with methionine deprivation for 6 h and methionine K) or SAM L) supplementation for 24h. Data were analyzed by unpaired two‐tailed Student's *t*‐test (A, B, C, D, G, I, and J) or Pearson *r* (H). Error bars denote for the s.e.m. ***p < 0.001.

SAM is the major methyl donor and is essential for nucleic acid and histone methylations, which are critical epigenetic modes that regulate gene transcription.^[^ ^38^ ^]^ Given the positive correlation between PCSK9 promoter methylation and its expression,^[^ ^39^ ^]^ it gives a hint that methionine catabolism may regulate the transcription of PCSK9 mRNA through DNA methylation. Indeed, supplementation of methionine increased the methylation of genomic DNA (Figure S4A, Supporting Information). Treating with 5‐azacytidine, the inhibitor of DNA methylation,^[^ ^40^ ^]^ disturbed the methionine‐ and SAM‐induced upregulation of *PCSK9* promoter activity (**Figure** **4**A,B; Figure S4B, Supporting Information). The levels of PCSK9 mRNA and protein induced by methionine and SAM were also inhibited by 5‐azacytidine (Figure 4C–F; Figure S4C,D Supporting Information). Nevertheless, the H3K4me3, a mark of active transcription,^[^ ^41^ ^]^ was decreased with methionine supplementation (Figure S4E, Supporting Information).

We next identified which DNA methyltransferase (DNMT) involved in the regulation of PCSK9 by methionine. DNMT1, an essential enzyme that maintains DNA methylation, was expressed at a higher level in colorectal cancer than in adjacent normal tissues (Figure 4G). And the expression of DNMT1 was strongly correlated with that of PCSK9 in tumor tissues (Figure 4H). Nevertheless, there are poor correlations between the expression of PCSK9 and the expressions of DNMT3A, DNMT3B, and DNMT3L (Figure S4F–H, Supporting Information). We further knocked down DNMT1 and detected the effect on PCSK9 expression (Figure S4I, Supporting Information). Knockdown of DNMT1 restrained the upregulation of *PCSK9* promoter activity and PCSK9 protein and mRNA expression induced by methionine or SAM (Figure 4I–L). Therefore, these combined results indicated that methionine catabolism promotes the mRNA transcription of PCSK9 through DNMT1‐mediated DNA methylation.

### 2.5 Methionine Inhibits Sirtuin 6 (SIRT6) Through DNA Methylation to Promote PCSK9 mRNA Transcription

**Figure 5:**
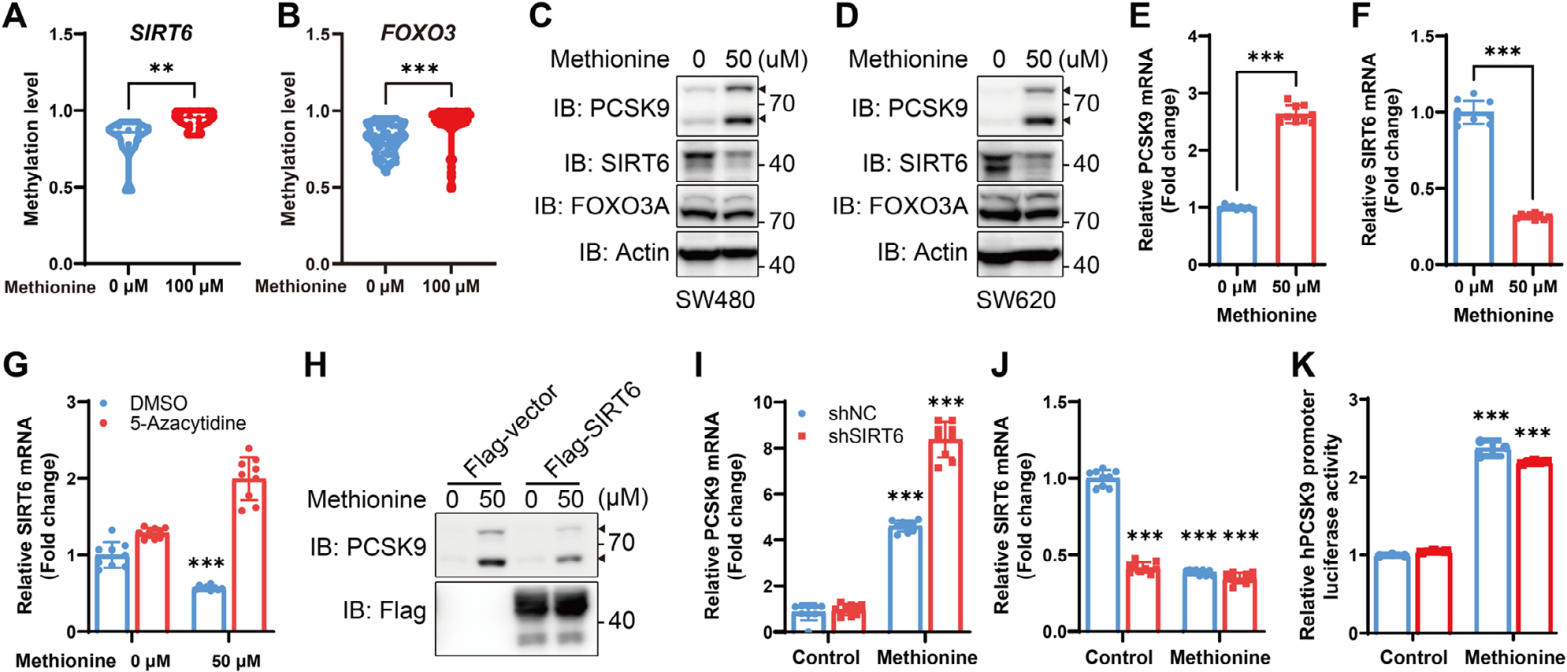
Methionine inhibits SIRT6 through DNA methylation to promote PCSK9 mRNA transcription. A,b) Methylation level of *SIRT6* A) and *FOXO3* B) in SW480 cells with methionine deprivation for 6 h followed by methionine supplementation for 6h. C,D) Immunoblotting analysis of PCSK9, SIRT6, and FOXO3A in SW480 (C) and SW620 (D) cells with methionine deprivation for 6 h and methionine supplementation for 24h. E,F) Relative mRNA level of PCSK9 (E, *n* = 9) and SIRT6 (F, *n* = 9) in SW480 cells with methionine deprivation for 6 h and then methionine supplementation (0, 50 µm) for 24h. G) Relative mRNA level of SIRT6 in SW480 cells with methionine deprivation for 6 h and then methionine supplementation together with 5‐azacytidine (5 µm) treatment for 24 h (*n* = 9). H) Immunoblotting analysis of PCSK9 and Flag in SW480 transfected with Flag‐SIRT6 and then cultured with methionine deprivation for 6 h and methionine supplementation for 24h. I,J) Relative mRNA level of PCSK9 (I) and SIRT6 J) and in SW480 cells transfected with shSIRT6 and culture with methionine deprivation for 6 h and then methionine supplementation for 24h. K) Luciferase reporter assays of hPCSK9 promoter activity in SW480 cells transfected with shSIRT6 (shNC served as a negative control) together with hPCSK9 promoter luciferase reporter plasmid and Renilla luciferase plasmid, and then cultured with methionine deprivation for 6 h followed by methionine supplementation for 24 h (*n* = 3). The relative hPCSK9 promoter luciferase activity for negative control was set as 1. Data were analyzed by unpaired two‐tailed Student's *t*‐test (A, B, E, F, G, I, J, and K). Error bars denote for the s.e.m. **p < 0.01, ***p < 0.001.

Given that inhibiting DNA methylation almost completely blocked the up‐regulation of PCSK9 induced by methionine (Figure 4), we conducted reduced representation bisulfite sequencing (RRBS), an efficient and high‐throughput technique for analyzing and comparing genomic methylation patterns on a single nucleotide level.^[^ ^42^ ^]^ We found that methionine supplementation increased the methylation level of most genes in different regions to a certain extent (Figure S5A,B, Supporting Information). Since DNA methylation mainly regulates gene expression by recruiting proteins involved in gene repression or by inhibiting the binding of transcription factors to DNA,^[^ ^43^ ^]^ and SIRT6 can be recruited by forkhead box O3 (FOXO3) and deacetylates histone H3 to suppress PCSK9 expression,^[^ ^44^ ^]^ we examined the DNA methylation of SIRT6 and FOXO3. The results showed that methionine supplementation improved the methylation level of both of them (**Figure** **5**A,B), but only decreased the protein level of SIRT6 (Figure 5C,D). The level of SIRT6 protein and mRNA showed an opposite trend to that of PCSK9 protein and mRNA when treated with methionine (Figure 5C‐F). Moreover, inhibition of DNA methylation by 5‐azacytidine promoted the mRNA transcription of SIRT6 (Figure 5G). These results implied the possibility that SIRT6 is involved in the regulation of PCSK9 by methionine. Furthermore, overexpression of SIRT6 partially inhibited the expression of PCSK9 induced by methionine (Figure 5H), while knocking down SIRT6 further enhanced the promotion of PCSK9 expression by methionine supplementation (Figure 5I,J). Nevertheless, the *PCSK9* promoter activity was not further improved when SIRT6 was knocked down (Figure 5K). Together these findings suggested that methionine inhibits SIRT6 through DNA methylation to promote PCSK9 mRNA transcription.

Besides, we analyzed the effect of methionine on methylation of PCSK9 promoter region using targeted bisulfite sequencing (Figure S5C, Supporting Information). Interestingly, the methylation level of *PCSK9* promoter region remained unchanged (Figure S5D, Supporting Information), but the methylation level of 4 gene body regions of *PCSK9* increased (Figure S5E,F, Supporting Information), indicating that it is not the promoter methylation but the gene body methylation of *PCSK9* that might participate in the regulation of its expression by methionine.

### 2.6 Dietary Methionine Restriction Potentiates PD‐1 Blockade Therapy for Microsatellite Stable (MSS) CRC

**Figure 6:**
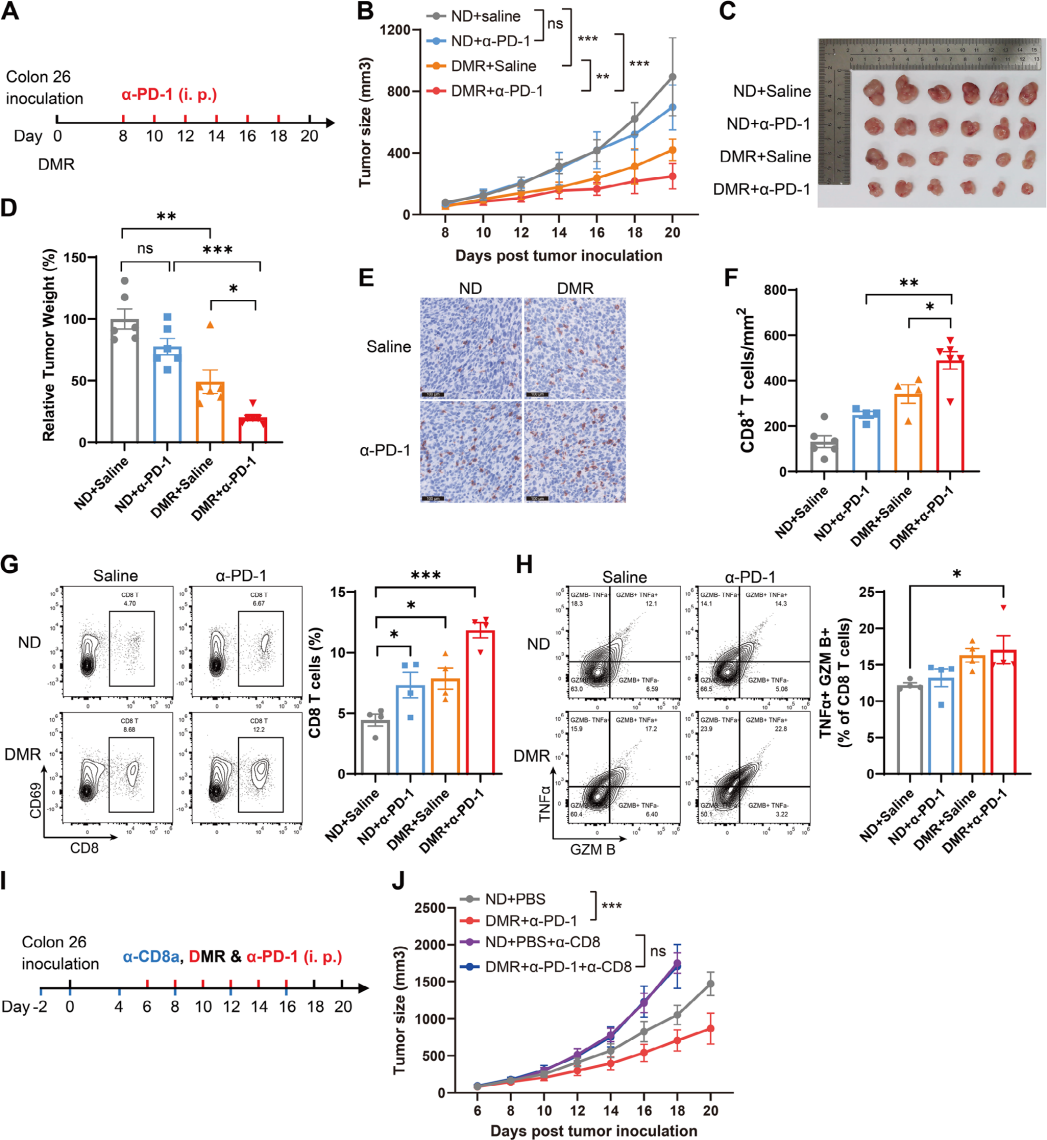
Dietary methionine restriction potentiates PD‐1 blockade therapy for MSS CRC. A) Schematic representation of combined therapy of PD‐1 blockade with dietary methionine restriction to Colon 26 tumor‐bearing mice. B–D) Tumor volume B), tumor size C), and relative tumor weight D) of Colon 26‐bearing mice with anti‐PD‐1 therapy and dietary methionine restriction (*n* = 6). E) Immunohistochemistry analysis of CD8 in tumor tissues from Colon 26‐bearing mice with anti‐PD‐1 therapy and dietary methionine restriction. F) Number of CD8 T cells in tumor tissues from Colon 26‐bearing mice with anti‐PD‐1 therapy and dietary methionine restriction was calculated (*n* = 4 or 6). G) Flow cytometric analysis of intratumoral CD8^+^ cytotoxic T cells (TILs) in Colon 26‐bearing mice fed with a methionine restriction diet alone or in combination with anti‐PD‐1 therapy. H) Flow cytometric analysis of TNFα and Granzyme B in intratumoral CD8^+^T cells from Colon 26‐bearing mice fed with a methionine restriction diet alone or in combination with anti‐PD‐1 therapy. I) Schematic representation of CD8 T cell depletion treatment in Colon 26 tumor‐bearing mice. J) Tumor volume of Colon 26‐bearing mice with anti‐PD‐1 therapy, dietary methionine restriction, and CD8 T cell depletion (*n* = 7 or 8). Data were analyzed by two‐way ANOVA B and J) or unpaired two‐tailed Student's *t*‐test (D, F, G, and H). Error bars denote for the s.e.m. ns, not significant; *p < 0.05, **p < 0.01, ***p < 0.001.

Immune checkpoint blockade therapy, especially the anti‐programmed cell death protein 1 (anti‐PD‐1) antibody, has emerged as an encouraging approach that changed the paradigm of cancer therapy.^[^ ^45^ ^]^ However, it has not yet shown a meaningful positive outcome for CRC patients with MSS/pMMR phenotype, representing 95% of metastatic CRC cases.^[^ ^46^ ^]^ Previous results demonstrated that DMR reduced PCSK9 expression and tumor growth in Colon 26‐bearing BALB/c mice (Figure 1; Figure S1, Supporting Information), a common MSS CRC tumor model resistant to anti‐PD‐1 therapy.^[^ ^47^ ^]^ However, whether DMR can improve the outcomes of anti‐PD‐1 therapy for MSS CRC remains unclear. To clarify this possibility, the Colon 26‐bearing BALB/c mice were treated with anti‐PD‐1 and/or DMR (**Figure** **6**A). Indeed, MSS Colon 26 syngeneic tumor models were non‐responsive to anti‐PD‐1 therapy, but the combination of DMR with anti‐PD‐1 therapy significantly enhanced the suppression of tumor progression compared to either DMR or anti‐PD‐1 therapy alone (Figure 6B–D). Similar findings were observed using MC38 syngeneic tumor model, a representative high microsatellite instability (MSI‐H) CRC tumor model (Figure S6A,B, Supporting Information).

Cytotoxic CD8^+^ T cells are the main effector cells in anti‐tumor immunity and constitute the cornerstone of cancer immunotherapy.^[^ ^48^ ^]^ Given that DMR enhances the potential of cancer immunotherapy, we next investigated whether CD8^+^ T cells are involved in the regulation of tumor progression by DMR. Immunohistochemistry and flow cytometry analyses demonstrated that DMR promoted the infiltration of intratumoral CD8+ cytotoxic T cells (TILs), and it was further enhanced by the combination of DMR with anti‐PD‐1 therapy (Figure 6E–G). Additionally, the effector function of TILs was improved in mice with the combination of DMR and anti‐PD‐1 therapy (Figure 6H). However, the measurement of T‐cell exhaustion and stemness markers showed no significant changes (Figure S6C, Supporting Information). Moreover, depletion of CD8^+^ T cells in vivo using an anti‐CD8 antibody significantly expedited tumor development in syngeneic immunocompetent mice (Figure 6I,J). However, there was no disparity in tumor development between a normal diet and a methionine restriction diet when CD8^+^ T cell depletion, indicating that DMR regulates tumor progression in a CD8^+^ T cell‐dependent manner (Figure 6J). Collectively, these results suggested that DMR promotes CD8^+^ T cell infiltration and potentiates anti‐PD‐1 therapy in MSS CRC, indicating that DMR may be a promising therapeutic approach for MSS CRC.

### 2.7 PCSK9 Inhibition Potentiates PD‐1 Blockade Therapy and 5‐Fluorouracil (5‐FU) Chemotherapy for MSS CRC

**Figure 7:**
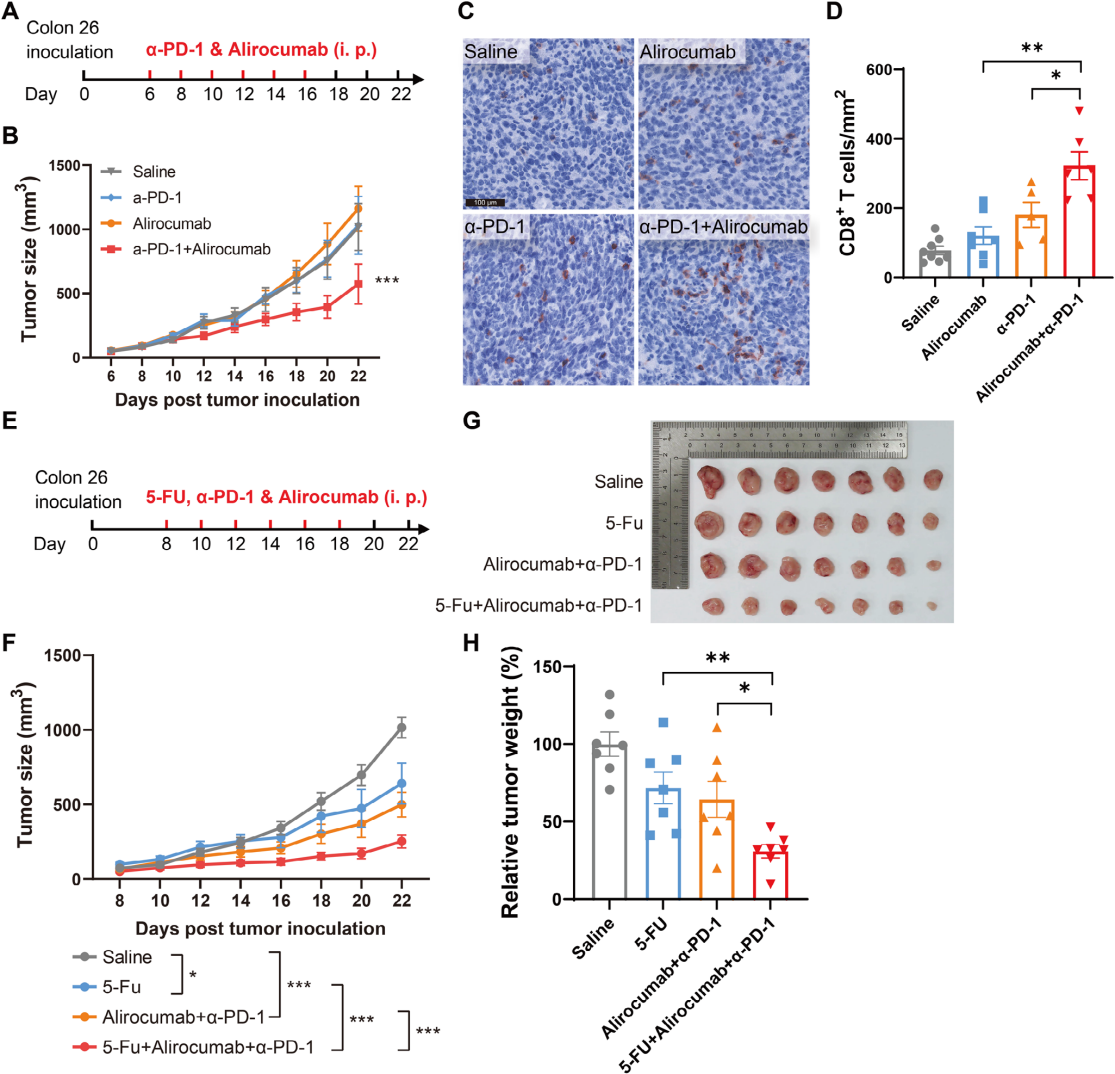
PCSK9 inhibition potentiates PD‐1 blockade therapy and 5‐FU chemotherapy for MSS CRC. A) Schematic representation of combined therapy of PD‐1 blockade with PCSK9 inhibition to Colon 26 tumor‐bearing mice. B) Tumor volume of Colon 26‐bearing mice with anti‐PD‐1 therapy and PCSK9 inhibition (*n* = 8 to 10). C) Immunohistochemistry analysis of CD8 in tumor tissues from Colon 26‐bearing mice with anti‐PD‐1 therapy and PCSK9 inhibition. D) Number of CD8 T cells in tumor tissues from Colon 26‐bearing mice with anti‐PD‐1 therapy and PCSK9 inhibition was calculated (*n* = 5 to 8). E) Schematic representation of combined therapy of 5‐FU chemotherapy with PD‐1 blockade and PCSK9 inhibition to Colon 26 tumor‐bearing mice. F–H) Tumor volume F), tumor size G), and relative tumor weight H) of Colon 26‐bearing mice with combined therapy of 5‐FU chemotherapy, PD‐1 blockade, and PCSK9 inhibition (*n* = 7). Data were analyzed by two‐way ANOVA (B and F) and unpaired two‐tailed Student's *t*‐test (D and H). Error bars denote for the s.e.m. *p < 0.05, **p < 0.01, ***p < 0.001.

While the significance of DMR in tumor progression is well established, how to render it feasible to achieve beneficial effects without inducing systemic toxicities in therapy remains a controversial topic.^[^ ^35^, ^49^ ^]^ Besides, the same regimen may lead to diverse outcomes depending on genetic and environmental factors.^[^ ^50^ ^]^ Previous results demonstrated that PCSK9, an encouraging target for cancer therapy, participates in regulating tumor progression via DMR (Figure 1I,J). To pursue an effective immunotherapy for MSS CRC tumor, we evaluated the effect of PCSK9 inhibition on MSS CRC tumor progression (**Figure** **7**A). The result showed that blocking PCSK9 with alirocumab, a clinically approved PCSK9 neutralizing antibody,^[^ ^51^ ^]^ did not halt MSS CRC tumor progression, akin to anti‐PD‐1 therapy (Figure 7B). However, the combination of PCSK9 inhibition with anti‐PD‐1 therapy significantly suppressed the tumor progression of MSS CRC (Figure 7B). Besides, the infiltration of CD8^+^ T cells in tumors was also increased upon this combined therapy, whereas it is not significant in the PCSK9 inhibition alone group (Figure 7C,D). Therefore, these findings suggested that PCSK9 inhibition restores the potency of anti‐PD‐1 for MSS CRC therapy, indicating that a combination of PCSK9 inhibition and anti‐PD‐1 therapy may be a feasible therapy strategy for MSS CRC.

Chemotherapy is the standard first‐line treatment for patients with metastatic CRC.^[^ ^52^, ^53^ ^]^ 5‐fluorouracil (5‐FU) is a fundamental component of chemotherapeutic agents for palliative and adjuvant treatments of CRC.^[^ ^54^, ^55^ ^]^ Therefore, we conducted a treatment that combined 5‐FU chemotherapy with PCSK9 inhibition and anti‐PD‐1 immunotherapy in the MSS CRC mouse model (Figure 7E). The results showed that the combination of PCSK9 inhibition and PD‐1 blockade improved the efficacy of 5‐FU chemotherapy for MSS CRC (Figure 7F–H). Besides, the effect of PCSK9 inhibition on tumor suppression in combination with 5‐FU treatment was also investigated (Figure S7A, Supporting Information). The data showed that PCSK9 inhibition alone also improved the efficacy of 5‐FU chemotherapy for MSS CRC (Figure S7B–D, Supporting Information).

Taken together, these observations suggested that PCSK9 inhibition restores the potency of anti‐PD‐1 and promotes the capability of 5‐FU for MSS CRC therapy, indicating two potential combination therapy regimens for MSS CRC, which is of great benefit to MSS CRC patients who account for 95% of metastatic CRC.^[^ ^46^ ^]^

## 3 Discussion

Nutrient metabolisms in the tumor microenvironment are interweavingly linked to tumor progression and immune evasion.^[^ ^1^, ^2^ ^]^ Amino acids, nutrients vital to the survival and proliferation of all cell types due to their roles in biosynthesis, energy metabolism, redox balance, and epigenetic modification,^[^ ^28^ ^]^ can also be converted into immunosuppressive metabolites such as kynurenine and spermidine, leading to tumor progression and immune evasion.^[^ ^10^, ^11^ ^]^ In addition, cholesterol, an indispensable component of most cellular membranes and central to all mammalian cells, however, is defective in tumor‐infiltrating CD8^+^ T cells.^[^ ^12^ ^]^ Loss of cholesterol leads to CD8^+^ T cell exhaustion and dysfunction.^[^ ^12^ ^]^ Moreover, an increase in plasma membrane cholesterol enhances the clustering of T‐cell antigen receptors and potentiates the anti‐tumor activities of CD8^+^ T cells.^[^ ^13^ ^]^ However, whether the nutrient metabolisms in tumor microenvironment can coordinate to regulate immune surveillance is unclear. Here we reported that methionine is catabolized to SAM and involved in DNA methylation to promote the expression of the key orchestrator of cholesterol metabolism, PCSK9, in colorectal cancer (Figure S8, Supporting Information). Our findings establish a mechanistic link between amino acid metabolism and cholesterol metabolism in the tumor microenvironment where tumor cells sense methionine to deploy PCSK9 expression.

PCSK9, a key cardiovascular disease target predominantly expressed in liver, is also aberrantly elevated in tumors, rendering it a promising candidate for cancer therapy.^[^ ^56^ ^]^ It is stringently regulated by various mechanisms at multiple levels, including SIRT6/FOXO3‐ and HINFP‐mediated epigenetic modifications,^[^ ^44^, ^57^ ^]^ SREBP2‐, HNF1α‐, E2F1‐, and SP1‐mediated mRNA transcription,^[^ ^32^, ^33^, ^58^, ^59^ ^]^ and miRNA‐mediated post‐transcriptional regulation.^[^ ^60^, ^61^ ^]^ In this study, we found that deprivation of methionine impedes the PCSK9 expression in CRC tumor tissue, without affecting its levels in the liver and blood. Methionine metabolism promotes the mRNA transcription of PCSK9 through DNA methylation and decreasing SIRT6. Our findings indicate a specific mechanism driving the abnormal elevation of PCSK9 expression in tumors through epigenetic regulation, which would provide a theoretical basis for targeting tumor PCSK9 expression in cancer immunotherapy.

While PD‐1/PD‐L1 blockade therapy has emerged as an encouraging approach for various cancers, it has not yet shown a meaningful positive outcome for MSS/pMMR CRC patients who account for 95% of metastatic CRC.^[^ ^46^ ^]^ The high expression of mismatch repair gene in MSS/pMMR CRC leads to a decrease in tumor neoantigens and epitopes, thereby resulting in poor immunogenicity.^[^ ^46^ ^]^ Additionally, the downregulation of MHC I further compromises antigen presenting,^[^ ^62^ ^]^ ultimately leading to the low infiltration and dysfunction of CD8^+^ T cells in MSS/pMMR CRC. Therefore, how to enhance CD8 T cell infiltration and function is the key to improve the sensitivity of MSS/pMMR CRC to immunotherapy. In this study, a combined therapy involving DMR with PD‐1 blockade was conducted in MSS CRC tumor model, and it was found that DMR promotes the infiltration and function of CD8^+^ T cells and potentiates PD‐1 blockade therapy by decreasing PCSK9 expression. Inhibition of PCSK9 also promotes the infiltration of CD8^+^ T cells and the sensitivity to PD‐1 blockade therapy. It is well established that PCSK9 is a key orchestrator of cholesterol metabolism, and abnormal cholesterol metabolism leads to poor immunogenicity or even immunosuppression, which is manifested by compromising the formation of peptide‐MHC II complex and impeding the clustering of T‐cell antigen receptors.^[^ ^13^, ^63^ ^]^ Furthermore, tumor cell‐derived PCSK9 hinders immune recognition by targeting MHC I for lysosome degradation^[^ ^20^ ^]^ and impairs anti‐tumor immunity by obstructing LDLR‐mediated TCR recycling and signaling in CD8 T cell.^[^ ^21^ ^]^ Therefore, inhibition of PCSK9 may improve the immunotherapeutic efficacy for MSS CRC from multiple dimensions: 1) promoting tumor cell antigen presentation by regulating MHC I and MHC II, 2) enhancing T cell cholesterol metabolism and 3) boosting T cell receptor signaling by improving LDLR‐mediated TCR recycling and TCR clustering, hence enhancing CD8^+^ T cell infiltration and function. Moreover, we found that combining 5‐FU chemotherapy with PCSK9 inhibition and PD‐1 blockade further enhances the therapeutic efficacy for MSS CRC. Our research reveals encouraged combination therapeutic approaches for MSS CRC and provides new insights into overcoming immunotherapy resistance.

Moreover, both dietary methionine restriction alone and a combination with PD‐1 blockade alleviated the tumor growth of MSS and MSI‐H CRC in mice. Nevertheless, knockdown of the immunometabolism checkpoint PCSK9 abolished the disparity in tumor progression induced by DMR. Together with the result that methionine promotes the mRNA transcription of PCSK9 through DNA methylation, our findings proposed a possibility that tumor cell methionine metabolism contributes to epigenetic regulation and immune responses linked to tumorigenesis. Indeed, dietary methionine restriction diminishes the YTH domain‐containing family protein 1 (YTHDF1)‐mediated N6‐methyladenosine (m6A) methylation and translation of immune checkpoints, including PD‐L1 and V‐domain Ig suppressor of T cell activation (VISTA),^[^ ^29^ ^]^ and boosts cyclic GMP‐AMP synthase (cGAS) activity by blocking SUV39H1‐mediated cGAS methylation,^[^ ^30^ ^]^ in tumor cells, thus alleviating tumor progression. In addition to its functions in tumor cells, methionine metabolism also facilitates the anti‐tumor activity of T cells by promoting histone methylation.^[^ ^6^ ^]^ Therefore, how to accurately target tumor methionine metabolism to inhibit tumor growth while preserving the anti‐tumor activity of T cells is worth further exploration. Previous studies have investigated the potential of targeting the highly expressed methionine transporter SLC43A2, the m6A‐specific binding protein YTHDF1, and the lysine methyltransferase SUV39H1 in tumor cells.^[^ ^6^, ^29^, ^30^ ^]^ Our results also suggest the feasibility of a combination therapy involving blockade of PD‐1 and inhibition of PCSK9, a novel effector of methionine catabolism and a key orchestrator of cholesterol homeostasis highly expressed in tumor cells, for MSS CRC. These findings would be conducive to comprehensively understanding the anti‐tumor mechanism of DMR and provide a theoretical foundation for developing clinically viable treatment strategies.

## 4 Experimental Section

### Plasmids and siRNAs

For PCSK9 gene promoter activity analysis, the human PCSK9 gene promoter (−900 bp to −1 bp) was cloned into pGL3 vector (Promega). To generate PCSK9 knockdown cell line, shRNA targeting mouse *Pcsk9* was cloned into pLKO.1 vector. The sequence of shPCSK9 was as follows: 5′‐GCTGATCCACTTCTCTACC‐3′. The sequence of shSLC43A2 was as follows: 5′‐GACCTTCGGTCCACGTTTATT‐3′. The sequence of shSIRT6 was as follows: 5′‐CAAGTGTAAGACGCAGTACGT‐3′. To generate PCSK9 knockout cell line, sgRNA targeting mouse *Pcsk9* was cloned into lenti‐CRISPR‐V2 vector. The sequence of sgRNA was as follows: 5′‐ACTTCAA‐CAGCGTGCCGG‐3′.

siRNAs were synthesized by Tsingke Biotech (Beijing, China). The sequences were as follows: si‐DNMT1‐1, 5′‐GGAACTTTGTCTCCTTCAA‐3′; si‐DNMT1‐2, 5′‐CAATGAGACTGACATCAAA‐3′; si‐SREBP2‐1, 5′‐CTGCAACAACAGACGGTAA‐3′; si‐SREBP2‐2, 5′‐GCCTCAGATCATCAAGACA‐3′; si‐SREBP2‐3, 5′‐CCTCTATTGGATGATGCAA‐3′; si‐HNF1A‐1, 5′‐GGTCCTACGTTCACCAACA‐3′; si‐HNF1A‐2, 5′‐CGAAGATGGTCAAGTCCTA‐3′; si‐HNF1A‐3, 5′‐CAGTGAGACTGCAGAAGTA‐3′.

### Reagents and Antibodies

For cell treatment, L‐methionine (HY‐N0326), SAH (HY‐19528), and 5‐azacytidine (HY‐10586) were from MedChemExpress (MCE), L‐Lysine monohydrochloride (L8662) and L‐Threonine (T8441) were from Sigma, SAM (S832588) was from Macklin, AG270 (T9050) was from TargetMol, actinomycin D (S8964) was from Selleck, and single amino acid deficient RPIM 1640 mediums were from Coolaber. For western blot analysis, antibodies to human PCSK9 (EPR7627(2)), DNMT1 (EPR18453), and H3K4me3 (ab8580) were from Abcam, antibody to mouse PCSK9 (AF3985) were from R&D Systems, antibody to Flag (F1804) was from Sigma‐Aldrich, antibody to β‐actin (C4) was from Santa Cruz, antibody to human SIRT6 (D8D12) and HRP‐conjugated secondary antibodies were from Cell Signaling Technology, antibody to human FOXO3A (A0102) was from Abclonal. For immunohistochemistry analysis, antibody to mouse PCSK9 (50251‐T26) was from Sino Biological, and antibody to mouse CD8 (98 941) was from Cell Signaling Technology. For DNA methylation assay, antibody to 5‐methylcytosine (D3S2Z) was from Cell Signaling Technology. For flow cytometry analysis, BV711 anti‐mouse CD69 (Clone H1.2F3, Cat No. 740 664), BV421 anti‐mouse Ly108 (Clone 13G3, Cat No. 740 090), BV650 anti‐mouse TIM3 (Clone RMT3‐23, Cat No. 755 162), fixation and permeabilization kit (554 714), and Transcription Factor Buffer Set (562 574) were from BD Bioscience, APC‐eFluor780 anti‐mouse CD45 (Clone 30‐F11, Cat No. 47‐0451‐82), APC anti‐mouse PD‐1 (Clone J43, Cat No. 17‐9985‐82), Super Bright 645 anti‐mouse PD‐1 (Clone J43, Cat No. 64‐9985‐82), APC‐eFluor 780 anti‐mouse CD8a (Clone 53–6.7, Cat No. 47‐0081‐82), APC anti‐mouse IFNγ (Clone XMG1.2, Cat No. 17‐7311‐82), PE anti‐mouse Granzyme B (Clone NGZB, Cat No. 12‐8898‐82), and LIVE/DEAD Fixable viability kits (L34964, L34994) were from Invitrogen, PE anti‐mouse CD8a (Clone 53–6.7, Cat No. 100 708) were from Biolegend, Alexa Fluor 488 anti‐mouse TCF1 (Clone C63D9, Cat No. 6444S) were from Cell Signaling Technology. For in vivo treatments, InVivoMab anti‐mouse PD‐1(RMP1‐14), anti‐CD8a antibody (2.43), and rat IgG2a (2A3) were from Bio X Cell, Alirocumab (Praluent) was from Sanofi, and 5‐Fluorouracil (S1209) was from Selleck. For in vivo dietary methionine restriction treatments, 0.86% methionine and 0.12% methionine diets were purchased from Dyets Company (Wuxi, China).

### Mice

C57BL/6 mice and BALB/c mice were provided by Zhiyuan (Guangzhou, China). Both sex and age‐matched mice (6–8 weeks) were used at the onset of the experiment. Mice were housed in individually ventilated cages under specific pathogen‐free conditions, at a temperature of 20–24 °C, a humidity of 40–70%, and a 12 h light/dark cycle from 7 am to 7 pm, with access to standard chow and autoclaved water. All animal experiments were performed according to the guidelines of the Research Ethics Committee of Guangdong Provincial People's Hospital (KY‐N‐2022‐079‐01).

### Tumor Inoculation and Treatments

For subcutaneous model, MC38 (1 × 10^6^) or colon26 (1 × 10^6^) were resuspended with 100 µL PBS and subcutaneously injected into the left flanks of C57BL/6 mice or BALB/c mice. From days 6 to 8, tumor size (volume) was measured every two days and calculated using the formula: tumor volume (mm^3^) = 0.52 × length × (width).^[^ ^2^ ^]^

For in vivo methionine restriction treatments, mice were housed on either a normal diet (ND) with 0.86% methionine or a dietary methionine restriction (DMR) diet with 0.12% methionine from the day after injection until the endpoints.

For therapy, tumor‐bearing mice with comparable tumor size were randomized into various groups to receive intraperitoneal injections of saline, anti‐PD‐1 antibody (RMP1‐14, Bio X Cell, 100 µg per injection), alirocumab (Sanofi, 500 µg per injection), or 5‐FU (Selleck, 25 mg kg^−1^, 600 ug per injection) as indicated groups on days 6, 8, 10, 12, 14, and 16 post tumor inoculation.

### Analysis of Intratumoral CD8^+^ Cytotoxic T Cell

Tumor‐bearing mice were anesthetized and sacrificed, tumor tissues were collected, weighed, and mechanically minced, and incubated in RPMI 1640 medium containing collagenase VI (250 U mL^−1^, Sigma), DNase I (50 U mL^−1^, Sigma) and hyaluronidase (0.25 mg mL^−1^, Sigma) for 20 min at 37 °C. The dissociated cells were passed through a 70 µm cell strainer (Biologix) and centrifuged at 50 ×*g* for 1 min. The supernatant was collected and centrifuged at 800 ×*g* for 10 min. The cells were then blocked with an anti‐CD16/32 antibody and stained with indicated surface antibodies for 30 min on ice. Dead cells were marked using live/dead fixable viability kits (ThermoFisher Scientific). Intracellular and/or transcription factor antibodies were added after fixation and permeabilization according to the manufacturer's instructions. For measurement of cytokine production, TILs were stimulated with PMA (50 ng mL^−1^) and ionomycin (1 µmol L^−1^) with BFA (5 µg mL^−1^) addition for 4 h at 37 °C. Flow cytometric data were acquired with a ThermoFisher Attune NxT flow cytometer and analyzed using FlowJo (version 10).

### Depletion of CD8^+^ T Cells In Vivo

To deplete CD8^+^ T cells, 200 µg of anti‐CD8α antibody (2.43, Bio X Cell) or rat IgG (2A3, Bio X Cell) were injected intraperitoneally 2 days before tumor inoculation and on days 0, 4, 8, 12, and 16 with tumor inoculation.

### PCSK9 Promoter Activity

SW480 and SW620 cells were seeded in 24‐well plates and transfected with pGL3‐hPCSK9 promoter‐luciferase plasmid (300 ng) together with Renilla (30 ng). After 6 h, the cells were treated with methionine‐depleted RPMI 1640 medium supplemented with methionine (50 µm) or SAM (500 µm) for an additional 24 h. Human PCSK9 gene promoter activity was analyzed using the Dual‐Luciferase Reporter Assay System Kit (E1910, Promega) according to the manufacturer's instructions.

### Real‐Time RT‐PCR

Total RNAs extracted with TRIzol reagent (Invitrogen, USA) were utilized for cDNA synthesis using HiScript III RT SuperMix for qPCR Kit (R323‐01, Vazyme) according to the manufacturer's instructions. cDNA samples were analyzed by real‐time PCR using 2× RealStar Fast SYBR qPCR Mix (A313, Genstar) on a CFX96 thermocycler system (Bio‐rad, USA). The housekeeping gene glyceraldehyde 3‐phosphate dehydrogenase (GAPDH) served as the internal control.

The gene‐specific primers (5′‐3′) were as follow: human PCSK9 (forward, GACGATGCCTGCCTCTACTC; reverse, CCAATGATGTCCTCCCCTGG), human SREBP2 (forward, ATCTGGATCTCGCCAGAGG; reverse, CCAGGCAGGTTTGTAGGTTG), human HNF1A (forward, GTGGCGAAGATGGTCAAGTCC; reverse, CCCTTGTTGAGGTGTTGGG), human SIRT6 (forward, GCCTGGTCATCGTCAACCTG; reverse, TCATGACCTCGTCAACGTAGC), human SLC43A2 (forward, CTGCTTCTTTAACTGGCCCC; reverse, CGTGGTCACCTGCTTGTAGA), human and mouse GAPDH (forward, CGGATTTGGTCGTATTGGGC; reverse, CGGTGCCATGGAATTTGCC).

### mRNA Stability Assay

To examine the impact of methionine supplementation on PCSK9 mRNA stability, SW480 and SW620 cells were treated with/without methionine, along with the transcription inhibitor actinomycin D (5 µg mL^−1^, Selleck, S8964), for 0, 6, and 12 h. Cells were collected for total RNA extraction and real‐time quantitative PCR analysis. The PCSK9 mRNA levels were normalized to baseline (0 h).

### Western Blotting

Tissues or cells were lysed in radioimmunoprecipitation assay (RIPA) buffer (Genstar) supplemented with protease inhibitor cocktail and phosphatase inhibitor cocktail (Selleck) on ice for 30 min and then centrifuged for 10 min at 12 000 rpm at 4 °C. The supernatant was collected and protein concentration was measured using a BCA Protein Assay Kit (TaKaRa). Proteins from each sample were separated by SDS‐PAGE, transferred to a PVDF membranes, probed overnight at 4 °C with primary antibodies, and followed by incubation for 1 h at room temperature with HRP‐conjugated secondary antibodies (Cell Signaling Technology). Signals were visualized by enhanced chemiluminescence (ECL; KeyGEN BioTECH) and imaged with an imaging system (Azure Biosystems, USA). Densitometry analysis was performed with AzureSpot Pro software.

### Immunohistochemistry

Tissue samples were fixed in 4% PFA for 24 h immediately after dissection, then processed for paraffin embedding and sectioned at 2.5 µm. Sections were deparaffinized, rehydrated, and subjected to antigen retrieval by heating in 10 mm sodium citrate (pH 6.0) using a high‐pressure cooker for 30 min, followed by blocking of endogenous peroxidase with 3% H_2_O_2_. Slides were blocked with goat serum and then incubated overnight at 4 °C with primary antibodies against PCSK9 (Sino Biological) and CD8 (Cell Signaling Technology) in a humidified chamber, followed by incubation with HRP‐conjugated secondary antibodies and visualization with 3‐amino‐9‐ethylcarbazole (AEC, A2010, Solarbio) and hematoxylin (Sigma Aldrich).

### Enzyme‐Linked Immunosorbent Assay (ELISA)

SW480 and SW620 cells were treated with methionine for 24 h, and the concentration of human PCSK9 in culture supernatants was determined by enzyme‐linked immunosorbent assay according to the manufacturer's instructions (SEK10594, Sino Biological). The concentration of mouse PCSK9 in serum was determined according to the manufacturer's instructions (SEK50251, Sino Biological). Briefly, A 96‐well plate (9018, Corning Costar) was coated overnight at 4 °C with monoclonal antibody to PCSK9. Triplicates of PCSK9 standards and cell culture supernatants or serum were then added to the plate and incubated for 2 h at room temperature. A biotinylated polyclonal antibody to PCSK9 was added to the plate, incubated for 1 h at room temperature, and followed by Avidin‐HRP addition and a 30 min incubation at room temperature. The amount of bound PCSK9 was assessed with TMB substrate and the reaction was terminated with 2N H2SO4. The absorbance of each well at 450 and 570 nm was then measured with a spectrophotometric plate reader (Synergy H1, Agilent).

### DNA Methylation

Genomic DNA was isolated using QIAamp DNA Mini Kit (51 304, QIAGEN) according to manufacturer's instructions and quantified using Nanodrop one (Thermo Fisher Scientific). Serially diluted DNA samples were prepared with a final concentration of 0.1 m NaOH, denatured at 95 °C for 10 min, and then chilled on ice immediately. The DNA samples were loaded to the nitrocellulose filter membrane (HATF00010, Merck Millipore). After UV‐crosslinking, the membrane was blocked with 5% non‐fat milk (in 1 × PBS with 0.05% Tween 20) for 1 h and incubated overnight at 4 °C with a specific anti‐m5C antibody. The membrane was further incubated with HRP‐conjugated secondary antibody (Cell Signaling Technology, 1:20 000) for 1 h at room temperature. Signals were visualized by enhanced chemiluminescence (ECL; KeyGEN BioTECH) and imaged with an imaging system (Azure Biosystems, USA). To ensure equal spotting of DNA onto the membrane, the same blot was stained with 0.02% methylene blue in 0.3 m sodium acetate at room temperature.

### Reduced Representation Bisulfite Sequencing and Targeted Bisulfite Sequencing

Genomic DNA was extracted using QIAamp DNA Mini Kit (51 304, QIAGEN) according to the manufacturer's protocol. For RRBS library preparation, 100 ng of genomic DNA was digested with MspI (New England Biolabs, USA), end‐repaired, 3′‐dA‐tailed, and ligated to 5‐methylcytosine‐modified adapters. The Adapter‐equipped DNA was subjected to sodium bisulfite conversion using EZ DNA Methylation‐Lightning Kit (Zymo Research, USA) according to the manufacturer's recommendations. After bisulfite treatment, the DNA was amplified with 12 cycles of PCR using Illumina 8‐bp dual index primers. Size selection was performed to obtain DNA fractions of MspI‐digested products in the range of 100–350 bp using a dual‐SPRI protocol according to the manufacturer's protocol. For targeted bisulfite sequencing libraries preparation, 200 ng of genomic DNA was converted using EZ DNA Methylation‐Lightning Kit (Zymo Research, USA), and the products were used as templates for PCR amplification using PyroMark PCR Kit (QIAGEN). BSP primers were designed using PyroMark Assay Design (v 2.0). The primers (5′‐3′) were as follow: PCSK9pro‐Primer1‐F: TAATTTTGGATTTTAGTATTTAGATTTAGAGTAGG; PCSK9pro‐Primer1‐R: CCTCAAATTATACATATAACATAACATAAAACTTA; PCSK9pro‐Primer2‐F: AATAGGTGTTTATTGGATGTTTGTTTAG; PCSK9pro‐Primer2‐R: CCTACATACATTTCAAAAAATTTATACTACAAAAATTC; PCSK9pro‐Primer3‐F: TAGTTAGTTGGTAAGGTTAGTGTGTA; PCSK9pro‐Primer3‐R: TTCTAAATCAATCCTACTATAAACTCCT; PCSK9pro‐Primer4‐F: GTAGGAGATAAGGTGGATTTAGGAAA; PCSK9pro‐Primer4‐R: AAACCCCCRAACTAAAAAATCAAATTTC; PCSK9pro‐Primer5‐F: TTTGAGTTTGGAGGAGTGAGTTAGGT; PCSK9pro‐Primer5‐R: ACTATCCTAACRAAAAAACCTAAAAACC. The PCR products were then end‐repaired, 3′‐dA‐tailed, and ligated to 5‐methylcytosine‐modified adapters. The constructed RRBS libraries and TBS libraries were then analyzed by Agilent 2100 Bioanalyzer and finally sequenced on Illumina platforms (NovaSeq 6000 PE150).

Adapter and barcode sequences were removed using Trimmomatic (v 0.3.6). The trimmed reads were mapped to the human genome version hg19 using BSMAP (v 2.7.3). After mapping to genome and generating SAM file, SAMTOOLS (version 1.9) was used to convert SAM file to BAM file, sort, and index for subsequent analysis. Methylation levels at individual sites were calculated using BSMAP (v 2.7.3). Differentially methylated regions were analyzed using metilene (v 0.2‐8) software.

### Statistical Analysis

TCGA gene expression was acquired via UCSC Xena browser (https://xenabrowser.net/) on Feb. 12, 2023. The PCSK9, MAT2A, AHCY, DNMT1, DNMT3A, DNMT3B, and DNMT3L expression data of primary solid tumor samples were analyzed by R software (R‐4.2.0‐win). Statistical analysis was performed using GraphPad Prism (version 9.0) software. Two‐way ANOVA was used for multiple comparisons in tumor growth delay experiments. Pearson correlation coefficient (*r*) was used to measure the linear correlation between gene expression. In other experiments, comparisons between two groups were made with unpaired two‐tailed Student's *t*‐tests. The data are presented as the means ± s.e.m. unless otherwise stated. *p*‐values of <0.05 were considered statistically significant.

## Conflict of Interest

The authors declare no conflict of interest.

## Author Contributions

Q.‐L.W., Z.C., and X.L. contributed equally to this work. This study was conceived and supervised by W.Y., J.Z., T.C., and Y.L.; research experiments were designed by W.Y. and Q.‐L.W., and conducted by Q.‐L.W., Z.C., X.L., H.L., H.F., N.W., L.C, M.L., L.L., L.H., and Y.D.; K.Z. and X.Z. participated in data analysis and interpretation; W.Y., Q.‐L.W., and Z.C. wrote the manuscript.

## Supporting information



Supporting Information

## Data Availability

The data that support the findings of this study are available from the corresponding author upon reasonable request.
