# Supplementary material for: Methionine Metabolism Dictates PCSK9 Expression and Antitumor Potency of PD‐1 Blockade in MSS Colorectal Cancer
Source: Adv Sci (Weinh). 2025 Mar 24;12(19):2501623. doi: 10.1002/advs.202501623 (PMC12097065; doi:10.1002/advs.202501623)
Supplement: Supplementary file 1 — Supporting Information [file ADVS-12-2501623-s001.docx]

Supporting Information

Methionine metabolism dictates PCSK9 expression and antitumor potency of PD-1 blockade in MSS colorectal cancer

Qi-Long Wang^#^, Zijie Chen^#^, Xiaofei Lu^#^, Huizhen Lin, Huolun Feng, Nuozhou Weng, Liwen Chen, Mengnan Liu, Li Long, Lingjun Huang, Yongmei Deng, Kehong Zheng, Xiaojun Zheng, Ting Cai*, Jiabin Zheng*, Wei Yang*


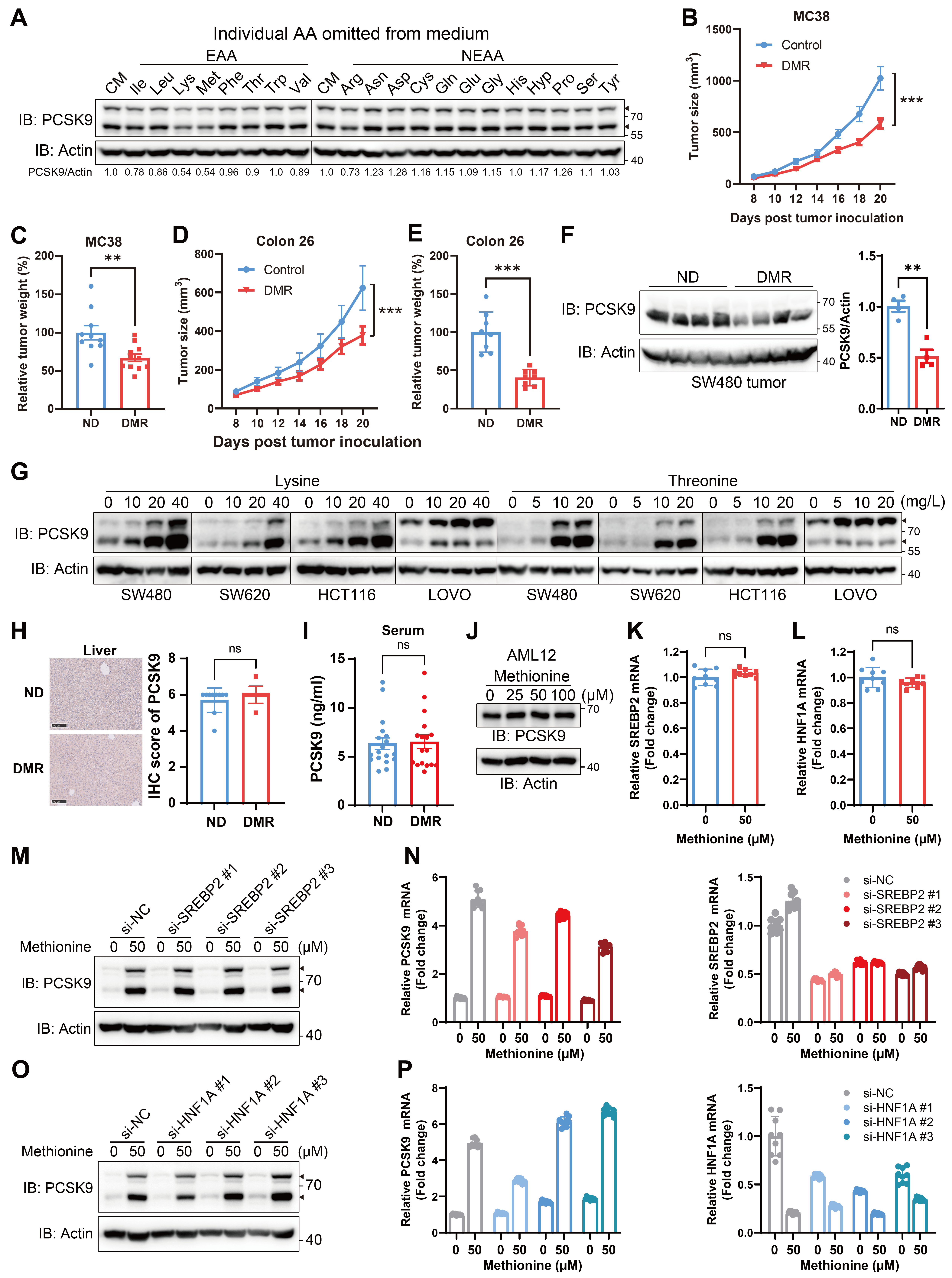


**Figure S1.** Methionine deprivation inhibits PCSK9 expression.

A) Immunoblotting analysis of PCSK9 in SW620 cells cultured with RPMI 1640 medium without individual amino acids for 6h. The densitometric quantification of the ratio of PCSK9 to β-actin is calculated. B,C) Tumor volume (B) and relative tumor weight (C) of MC38-bearing mice fed with normal diet (n = 10) and methionine restriction diet (n = 11). D,E) Tumor volume (D) and relative tumor weight (E) of Colon 26-bearing mice fed with normal diet (n = 8) and methionine restriction diet (n = 8). F) Immunoblotting analysis of PCSK9 in tumors from SW480-bearing *Rag2*-/- mice fed with normal diet and methionine restriction diet. The densitometric quantification of the ratio of PCSK9 to β-actin is calculated. G) Immunoblotting analysis of PCSK9 in human CRC cell lines (SW480, SW620, HCT116, and LoVo) with lysine or threonine deprivation for 6h and then lysine or threonine supplementation for 24h. H) Immunohistochemistry analysis of PCSK9 in livers from MC38-bearing mice fed with normal diet and methionine restriction diet (left). The abundance of PCSK9 was assessed (right). I) PCSK9 levels in serum from MC38-bearing mice fed with normal diet (n = 18) and methionine restriction diet (n = 17). J) Immunoblotting analysis of PCSK9 in mouse liver cells (AML12) with methionine deprivation for 6h and then methionine supplementation for 24h. K,L) Relative mRNA level of SREBP2 (K) and HNF1A (L) in AML12 cells with methionine deprivation for 6h and then methionine supplementation for 24h. M,N) Immunoblotting analysis of PCSK9 (M) and relative PCSK9 and SREBP2 (N) expression in SW480 transfected with si-SREBP2 (si-NC served as a negative control), and then cultured with methionine deprivation for 6h and methionine supplementation for 24h. O,P) Immunoblotting analysis of PCSK9 (O) and relative PCSK9 and HNF1A (P) expression in SW480 transfected with si-HNF1A (si-NC served as a negative control), and then cultured with methionine deprivation for 6h and methionine supplementation for 24h. Data were analyzed by two-way ANOVA (B and D) or unpaired two-tailed Student’s *t*-test (C, E, H, I, K, and L). Error bars denote for the s.e.m. ns: not significant; **P < 0.01, ***P < 0.001.


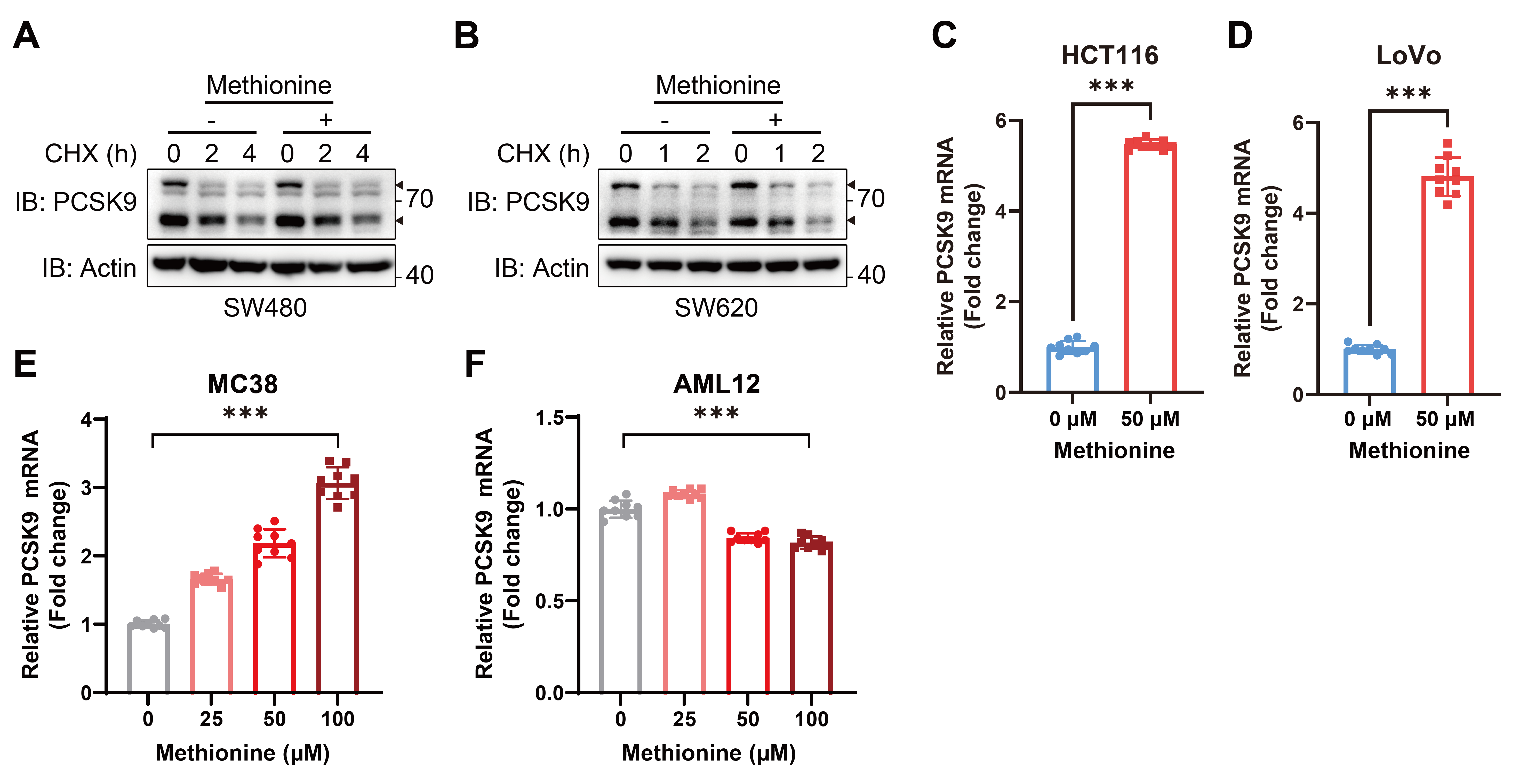
**Figure S2.** Methionine promotes PCSK9 mRNA transcription.

A,B) Immunoblotting analysis of PCSK9 in SW480 (A) and SW620 (B) cells cultured with or without methionine and treated with cycloheximide (50 μg/ml) for the indicated time. C-F) Relative mRNA level of PCSK9 in HCT116 (C, n = 9), LoVo (D, n = 9), MC38 (E, n = 9) and AML12 (F, n = 9) cells with methionine deprivation for 6h and then methionine supplementation for 24h. Data were analyzed by unpaired two-tailed Student’s *t*-test (C, D, E, and F). Error bars denote for the s.e.m. ***P < 0.001.

**Figure S3.** Methionine is catabolized to SAM to promote PCSK9 expression.

A,B) Immunoblotting analysis of PCSK9 in SLC43A2-KD SW480 (A) and SW620 (B) cells with methionine deprivation for 6h and then methionine supplementation for 24h (left). Relative mRNA level of SLC43A2 in SW480 (A) and SW620 (B) cells transfected with sh-SLC43A2 were shown (right, n = 9). C) The mRNA level of AHCY in tumor tissues (n = 476) and normal tissues (n = 41) of Colon Adenocarcinoma from TCGA database. D) Correlation between AHCY and PCSK9 mRNA levels in tumor tissues (n = 476) of Colon Adenocarcinoma from the TCGA database was calculated using linear regression. E,F) Immunoblotting analysis of PCSK9 in HCT116 (upper panel) and LoVo (lower panel) cells with methionine deprivation for 6h and then SAM (E) or SAH (F) supplementation for 24h. Data were analyzed by unpaired two-tailed Student’s *t*-test (A, B, and C) or pearson *r* (D). Error bars denote for the s.e.m. ***P < 0.001.


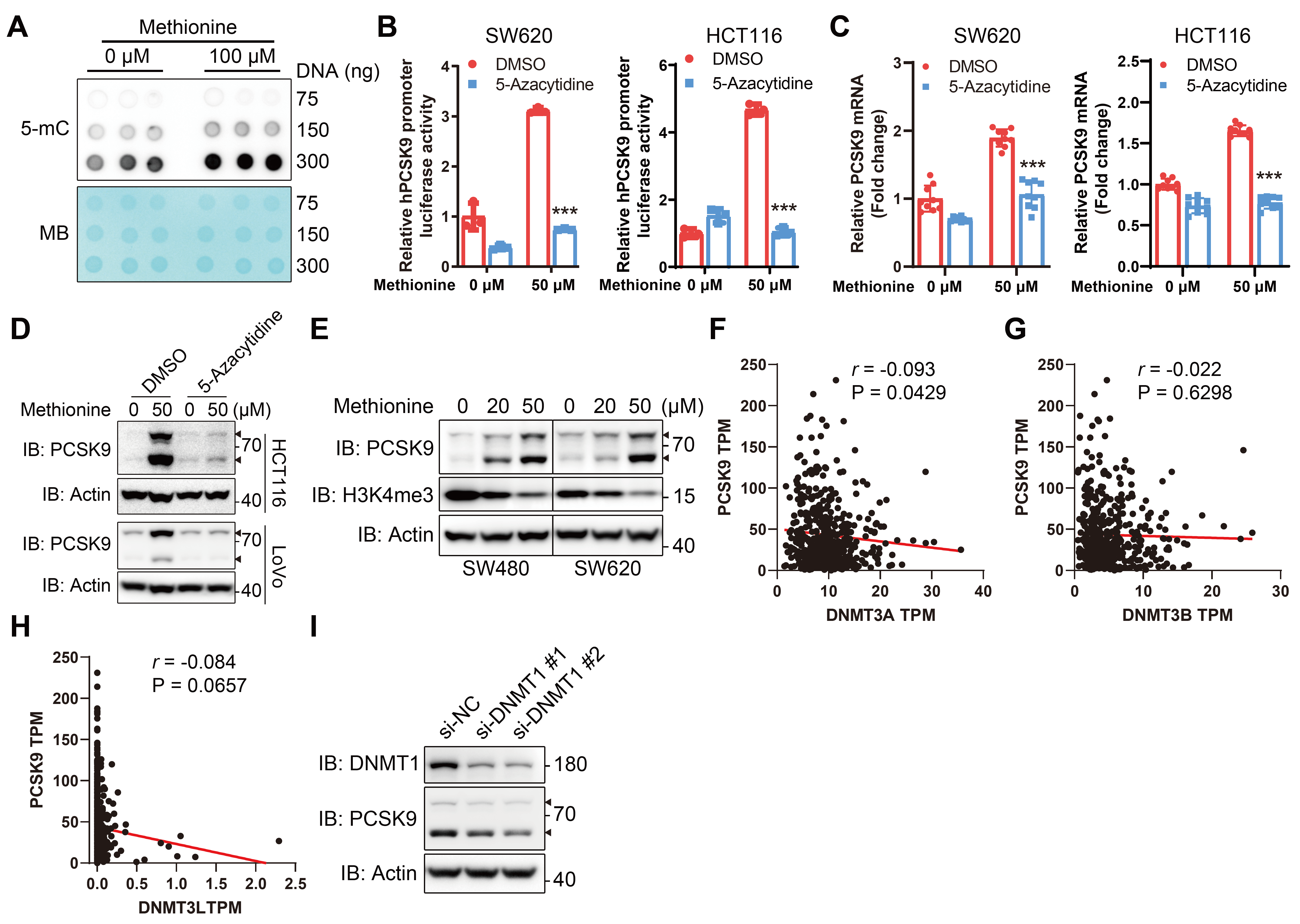
**Figure S4.** DNA methylation promotes mRNA transcription of PCSK9.

A) Immunoblotting analysis of DNA methylation in SW480 cells cultured with or without methionine for 6h. B) Luciferase reporter assays of hPCSK9 promoter activity in SW620 (left) and HCT116 (right) cells transfected with hPCSK9 promoter luciferase reporter plasmid together with Renilla luciferase plasmid, and then cultured with methionine deprivation for 6h followed by methionine supplementation and 5-azacytidine (5 μM) treatment for 24h (n = 3). The relative hPCSK9 promoter luciferase activity for methionine starvation group was set as 1. C) Relative mRNA level of PCSK9 in SW620 (left) and HCT116 (right) cells with methionine deprivation for 6h and then methionine supplementation together with 5-azacytidine (5 μM) treatment for 24h (n = 9). D) Immunoblotting analysis of PCSK9 in HCT116 (upper panel) and LoVo (lower panel) cells with methionine deprivation for 6h, and then methionine supplementation together with 5-azacytidine (5 μM) treatment for 24h. E) Immunoblotting analysis of the methylation of histone H3 in SW480 and SW620 cells with methionine deprivation for 6h and then methionine supplementation for 24h. F-H) Correlation between PCSK9 and DNMT3A (F), DNMT3B (G), and DNMT3L (H) mRNA levels in tumor tissues (n = 476) of Colon Adenocarcinoma from the TCGA database were calculated using linear regression. I) Immunoblotting analysis of PCSK9 and DNMT1 in SW480 cells transfected with si-DNMT1 for 24h. si-NC served as a negative control. Data were analyzed by unpaired two-tailed Student’s *t*-test (B and C) or pearson *r* (F, G, and H). Error bars denote for the s.e.m. ***P < 0.001.


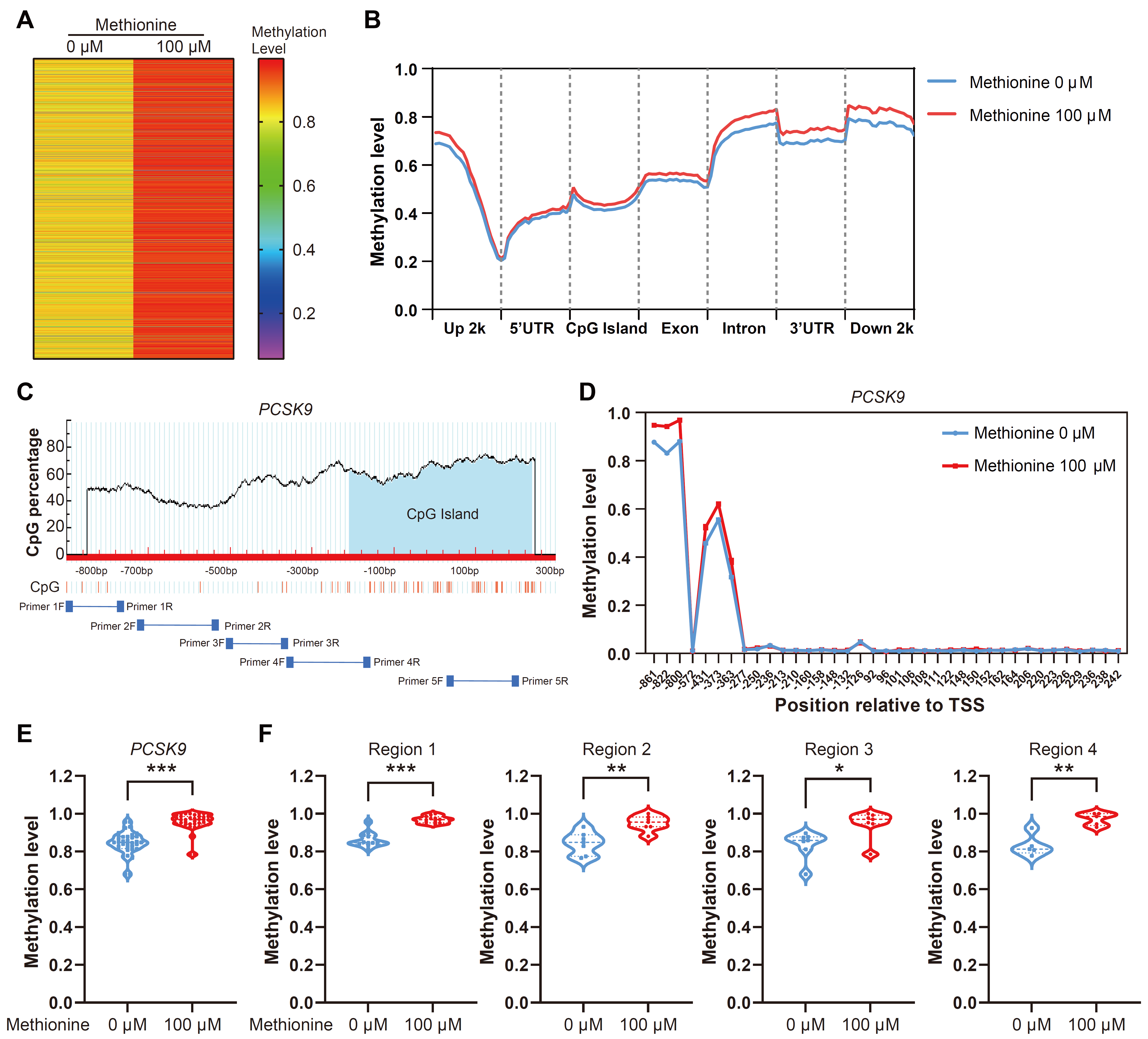
**Figure S5** Methylation profile in SW480 cells upon methionine treatment.

A) Methylation profile in SW480 cells with methionine deprivation for 6h and then methionine supplementation for 6h. B) Comparison of the mean methylation levels in various genomic regions between the methionine starvation and supplementation groups. C) Schematic representation of GpC percentage of the putative promoter regions of human PCSK9. Locations of the primer sets for the targeted bisulfite sequencing (TBS) are indicated. The promoter sequences of PCSK9 were obtained from the NCBI. CpG island prediction and design for methylated primers were conducted by using the Methprimer web server. D) Methylation profile of PCSK9 promoter region acquired by TBS. E,F) Methylation levels of PCSK9 gene body regions acquired by RRBS. Data were analyzed by unpaired two-tailed Student’s *t*-test (E and F). Error bars denote for the s.e.m. *P < 0.05, **P < 0.01, ***P < 0.001.


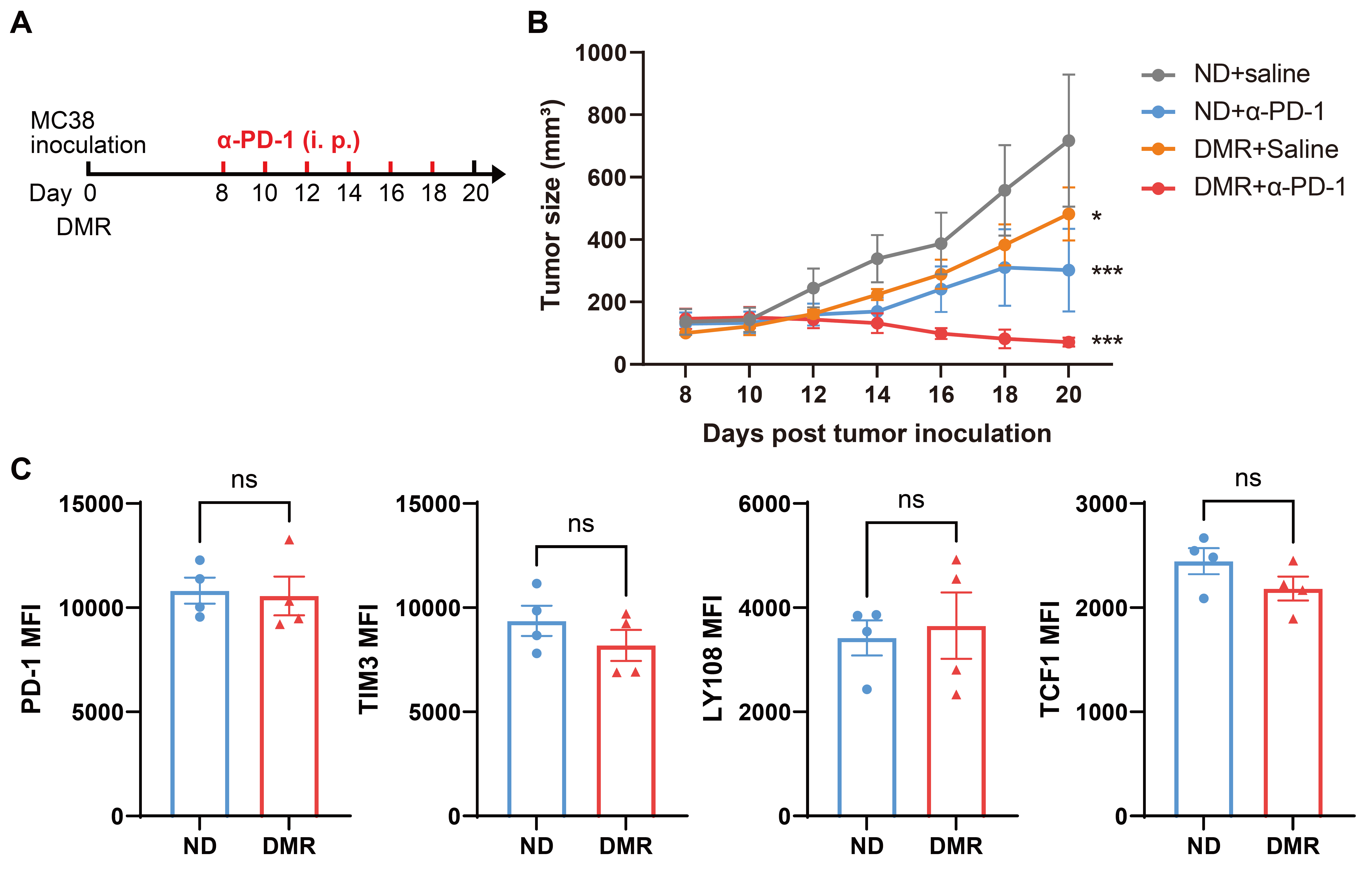
**Figure S6.** Dietary methionine restriction potentiates PD-1 blockade therapy for MSI-H CRC.

A) Schematic representation of combined therapy of PD-1 blockade with dietary methionine restriction to MC38 tumor-bearing mice. B) Tumor volume of MC38-bearing mice with anti-PD-1 therapy and dietary methionine restriction (n = 5). C) Flow cytometric analysis of PD-1, TIM3, LY108, and TCF1 in intratumoral CD8^+^ T cells from Colon 26-bearing mice fed with a normal diet and a methionine restriction diet. Data were analyzed by two-way ANOVA (B) or unpaired two-tailed Student’s *t*-test (C). Error bars denote for the s.e.m. ns, not significant; *P < 0.05, ***P < 0.001.

**Figure S7.** PCSK9 inhibition potentiates 5-FU chemotherapy for MSS CRC.

A) Schematic representation of combined therapy of 5-FU chemotherapy with PCSK9 inhibition to Colon 26 tumor-bearing mice. B-D) Tumor volume (B), tumor size (C), and relative tumor weight (D) of Colon 26-bearing mice with combined therapy of 5-FU chemotherapy and PCSK9 inhibition (n = 6). Data were analyzed by two-way ANOVA (B) or unpaired two-tailed Student’s *t*-test (D). Error bars denote for the s.e.m. *P < 0.05, **P < 0.01, ***P < 0.001.

**Figure S8.** Schematic illustration of the regulatory role of methionine catabolism on PCSK9 expression in tumor cells.

Methionine is catabolized to S-adenosyl-methionine (SAM) to promote PCSK9 transcription via DNMT1-mediated DNA methylation and suppressing SIRT6 expression, leading to the ectopic elevation of PCSK9 expression and tumor immunotherapy resistance.
